# Warm Body Temperature Facilitates Energy Efficient Cortical Action Potentials

**DOI:** 10.1371/journal.pcbi.1002456

**Published:** 2012-04-12

**Authors:** Yuguo Yu, Adam P. Hill, David A. McCormick

**Affiliations:** 1 Department of Neurobiology and Kavli Institute for Neuroscience, Yale University School of Medicine, New Haven, Connecticut, United States of America; 2 Center for Computational Systems Biology, Fudan University, Shanghai, People's Republic of China; Indiana University, United States of America

## Abstract

The energy efficiency of neural signal transmission is important not only as a limiting factor in brain architecture, but it also influences the interpretation of functional brain imaging signals. Action potential generation in mammalian, versus invertebrate, axons is remarkably energy efficient. Here we demonstrate that this increase in energy efficiency is due largely to a warmer body temperature. Increases in temperature result in an exponential increase in energy efficiency for single action potentials by increasing the rate of Na^+^ channel inactivation, resulting in a marked reduction in overlap of the inward Na^+^, and outward K^+^, currents and a shortening of action potential duration. This increase in single spike efficiency is, however, counterbalanced by a temperature-dependent decrease in the amplitude and duration of the spike afterhyperpolarization, resulting in a nonlinear increase in the spike firing rate, particularly at temperatures above approximately 35°C. Interestingly, the total energy cost, as measured by the multiplication of total Na^+^ entry per spike and average firing rate in response to a constant input, reaches a global minimum between 37–42°C. Our results indicate that increases in temperature result in an unexpected increase in energy efficiency, especially near normal body temperature, thus allowing the brain to utilize an energy efficient neural code.

## Author Summary

Conserving energy is essential to life. The brain, while only 2% of the body mass, uses an astounding 20% of its energy. It has long been assumed that this large energy consumption was due to the need to generate the electrical signals through which brain cells communicate: the action potentials. However, recent results reveal that the wires of the mammalian brain – the axons – are remarkably energy efficient. How is this energy efficiency obtained? Here we addressed this question by performing recordings and computational models of mammalian brain cells. We found that the increase in body temperature associated with the evolution of warm-blooded animals had an energetic benefit. The action potentials of warm-blooded animals became remarkably energy efficient, owing simply to the increase in body temperature. These results indicate that mammalian brains, although requiring a great deal of energy to operate, are actually more efficient than expected.

## Introduction

Brain signaling is metabolically expensive. Energy expenditure not only constrains the size and architecture of the brain, which limits its computational power, but is critical to the interpretation of functional brain imaging signals through related metabolic mechanisms (e.g. oxygen consumption and blood flow) [1]. Comprising only about 2% of the body's mass, the mammalian brain consumes about 20% of its energy [2], [3], [4]. Another unique feature of mammals is their warm body temperature (about 35–39°C). How a warm body temperature affects signaling and energy budget in the brain is largely unknown. Here we address this critical and interesting question through simple Hodgkin-Huxley models as well as recordings from cortical neurons during changes in temperature. Operating neurons is expensive, in part owing to the need to maintain a significantly higher concentration of Na^+^ ions outside, versus inside, nerve cells [5], [6], [7], [8]. Na^+^ entry into neurons, which must be returned to its extracellular location through the operation of the Na^+^/K^+^ ion pump by the expenditure of energy via hydrolysis of ATP, occurs through generation of action potentials (particularly along long intracortical, unmyelinated axons) and synaptic potentials during active signaling. These influxes of Na^+^ into neurons occur in addition to a background leak of Na^+^ ions through the neuronal membrane. Thus, to understand the energy costs of neuronal signaling in the cortex, it is essential to understand the entry of Na^+^ into neurons and neuronal processes during neuronal activity.

A maximally energy efficient action potential would entail no overlap of the inward Na^+^ current that generates the upstroke and the outward K^+^ current that facilitates the downstroke. Any overlap of these two opposing currents would merely result in an electrically neutral exchange of positive ions. Classic investigations by Hodgkin of squid giant axon revealed an excess entry of approximately 4 times as much Na^+^ as minimally required to generate the action potential [9]. This value of 4 times excess Na^+^ entry has figured prominently in estimates of the distribution of the sources of energy consumption in the mammalian brain [1], [5], [10], [11], as well as in the calculation of the average firing rate of cortical neurons [6]. For example, one classic modeling study of energy consumption in mammalian brains stated “A realistic estimate of the Na^+^ entry needed is obtained [for action potential generation] by quadrupling [the minimal Na^+^ entry] to take account of simultaneous activation of Na^+^ and K^+^ channels” [5]. Calculations such as these have predicted that up to 50% or more of the energy consumption in mammalian brains is devoted to the reversal of ion exchanges owing to Na^+^ entry (and K^+^ exit) during action potentials [5], [10]. Based upon this calculation, it has been proposed that the brain can support an average firing rate of less than 0.2 spikes/second, suggesting that the nervous system operates through a very sparse code [6].

Reconstruction of the inward Na^+^ and outward K^+^ currents occurring during action potential generation in mammalian cortical axons revealed, in contrast to the results predicted, an excess ratio of Na^+^ entry of only 1.3 [12], [13], indicating that axons in the mammalian brain are far more energy efficient than previously appreciated. This high energy efficiency in action potential generation is achieved through a relatively complete Na^+^ channel inactivation prior to substantial activation of the outward K^+^ current [12], [13]. These results prompted studies based on the hypothesis that the kinetics of ion channels may be optimized through evolution from invertebrates (e.g. squid giant axon) to mammals (e.g. rodent cortical axons) [12], [13]. This increased efficiency of mammalian axons has important implications not only for the average firing rate of cortical neurons, but also the practical limitations on the size and morphology of the brain. Here, we reexamine this issue through a set of computational models and experimental study. We hypothesize that the achievement of the highly energy efficient action potentials in cortical neurons is achieved in large part through the development of a warm body temperature.

Prior recording and modeling studies have demonstrated that the efficiency of action potential generation is highly sensitive to the kinetics of underlying ionic currents [13], [14], [15]. Indeed, changing temperature has a strong influence on the kinetics of the Na^+^ and K^+^ currents underlying action potential generation [16], [17], [18]. Homeothermic animals (e.g. mammals and birds) have the ability to maintain a relatively constant brain temperature in the range of 34–42°C [19], [20], [21], [22], [23], [24], while poikilothermic animals experience much wider variations in body temperature. The species of squid most studied (*Loligo*) lives in an ocean environment varying in temperature from approximately 10–23°C, which is far colder than naturally occurring in most mammals. Since temperature is a strong determinant of ion channel kinetics, which in turn can dramatically change action potential efficiency, we explored here the possibility that mammalian neurons may generate action potentials with maximal energy efficiency at normal body temperatures.

## Results

**Figure 1:**
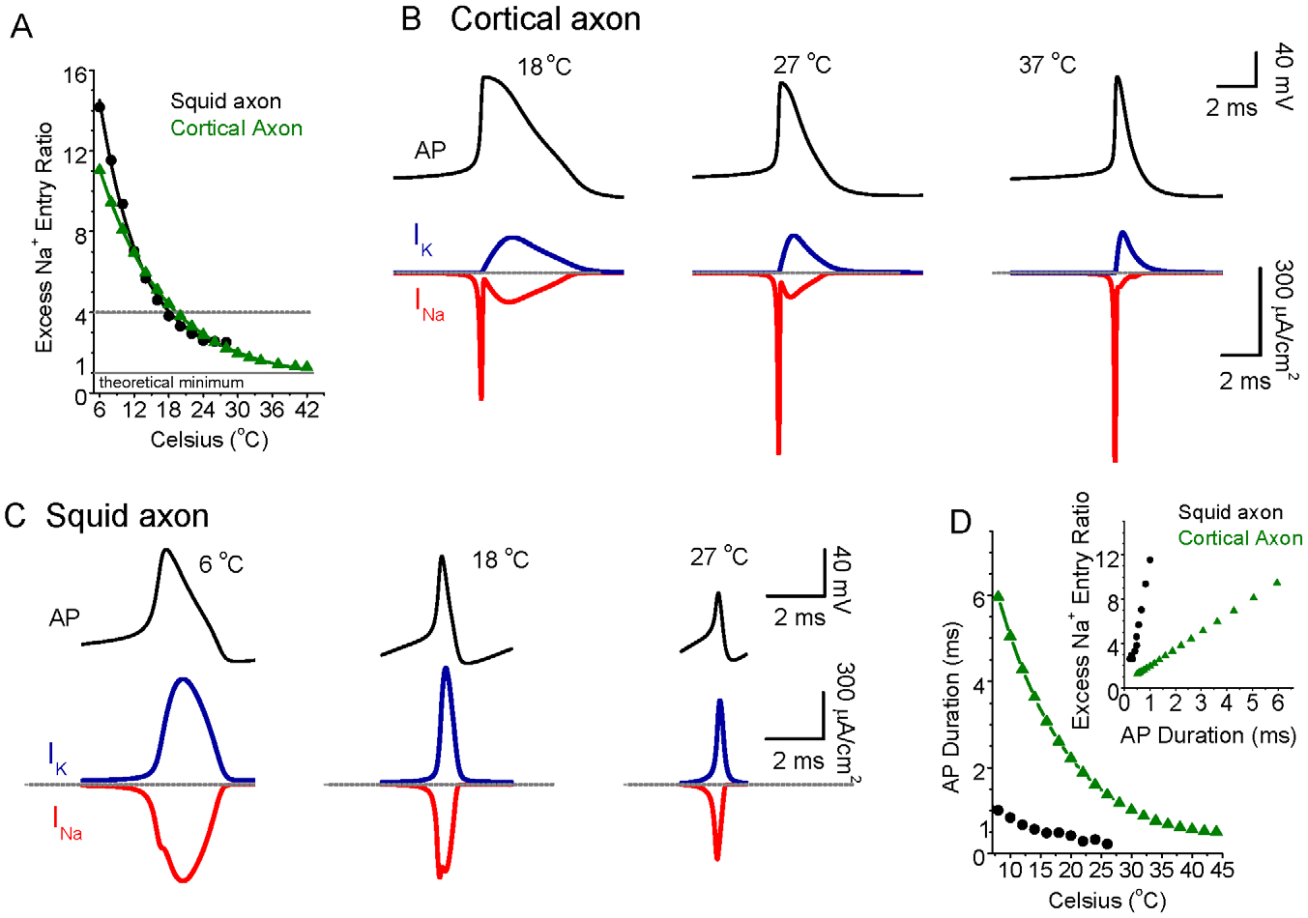
The energy efficiency of action potential generation increases as temperature increases. A. Na^+^ entry ratio (defined as a ratio of the total actual sodium entry to the minimal amount needed to generate an action potential, i.e., ∫I_Na_(t)dt/(C_m_ΔV), where I_Na_(t) is the sodium current, C_m_ is membrane capacitance, ΔV is the change in voltage during the action potential) as a function of temperature. For both the HH simulation of the squid giant axon (DC injection 20×10^−2^ pA/µm^2^), and for a model of cortical pyramidal cell axons (DC injection 0.5×10^−2^ pA/µm^2^), increasing temperature strongly decreases the excess Na^+^ entry during action potential generation. At 18°C, this entry ratio is approximately 4 (dashed line), while at 37°C, this excess entry reaches 1.89 (HH, extrapolated) and 1.41 (cortical axon), which is close to the theoretical minimum. B. Top panel: the action potential of the cortical model neuron generated at 18, 27 and 37°C, respectively; Bottom panel: the corresponding Na^+^ and K^+^ currents during action potential generation for temperature at 18, 27 and 37°C, respectively. Note that there is substantial overlap of Na^+^ and K^+^ currents during action potential generation for 18°C, reduced overlap at 27°C and much less overlap at 37°C. The largely non-overlapping nature of the ionic currents at high temperature results in considerably lower excess Na^+^ entry ratio. Dashed lines indicate the peak of each action potential for reference. C. Top panel: the action potential of classical HH model neuron generated at 6, 18, and 27°C, respectively; Bottom panel: the corresponding Na^+^ and K^+^ currents during action potential generation for temperature at 6, 18, and 27°C, respectively. Notice that the overlap of Na^+^ and K^+^ currents during action potential generation is also reduced when temperature increases. D. The half-height spike duration decreases as a function of temperature increase for both classical HH neuronal model (black) and cortical neuronal model (green). Inset: The correlation between excess Na^+^ entry ratio and spike duration for both models. The large spike duration corresponds to a large Na^+^ entry ratio, indicating reduced energy efficiency.

The examination of spike efficiency through simulation studies of squid giant axon action potential generation by Hodgkin [9] were performed with a temperature of 18°C, while the recordings of mammalian axons were performed at 37°C [12]. We explored whether or not these variations in temperature may help to explain the marked difference in excess Na^+^ entry between these two species by performing simple Hodgkin-Huxley style simulations of action potentials in either the traditional HH single compartment model or in a simple uniform cylindrical model of a cortical axon [25] and varying temperature (Figure 1A). We used simple, single compartment models with only I_Na_, I_K_, and I_Leak_ so as to clearly demonstrate the principles of the effects of temperature on excess Na^+^ entry during action potentials. Simulations with more complete computational models of layer 5 pyramidal neurons, including back propagating action potentials from the axon [25], yielded similar results as those reported here. For the present simulations, we assumed a Q_10_ for both Na^+^ and K^+^ currents of 2.3 [17], [18], [26], [27] and varied reversal potential with temperature according to the Nernst equation. Varying the Q_10_ used from 1.5 to 3 yielded qualitatively similar results as those shown in Figure 1. Using either the traditional HH or cortical axon single compartment models, increases in temperature caused a marked decrease in the excess Na^+^ entry occurring during action potential generation, such that at 18°C a value of approximately 4X excess is obtained, while at 37°C, a value of 1.41 is observed for our model of cortical axons (Figure 1A). The traditional HH model of the squid axon fails to generate action potentials at temperatures above approximately 28°C. At this temperature, the excess Na^+^ ratio is 2.5 (Figure 1A). In our model of a cortical axon spike, examination of action potentials at 18°C reveal an inward Na^+^ current that exhibits a prominent inward shoulder during the repolarizing phase of the spike, resulting in a strong overlap of inward Na^+^ and outward K^+^ currents (Figure 1B), as in squid giant axons at this temperature (Figure 1C; [9]). The same simulation, but at 37°C, however reveals an inward Na^+^ current that overlaps very little with the outward K^+^ current, as seen in mammalian axons at 37°C (Figure 1B; [12], [13]). For the squid axon HH model, the overlap of inward Na^+^ and outward K^+^ also decreases when temperature increases (Figure 1C). In addition, we also notice a dramatic decrease in spike duration as a function of temperature for both models (see Figure 1D). There is a nearly linear correlation between spike duration and excess Na^+^ entry ratio (see Figure 1D, inset). This relationship in our models arises from the fact that lowering temperature results in kinetically slower ionic currents, resulting in both an increase in overlap of the inward Na^+^ and outward K^+^ currents and a longer duration action potential (see Figure 1B, D).

**Figure 2 pcbi-1002456-g002:**
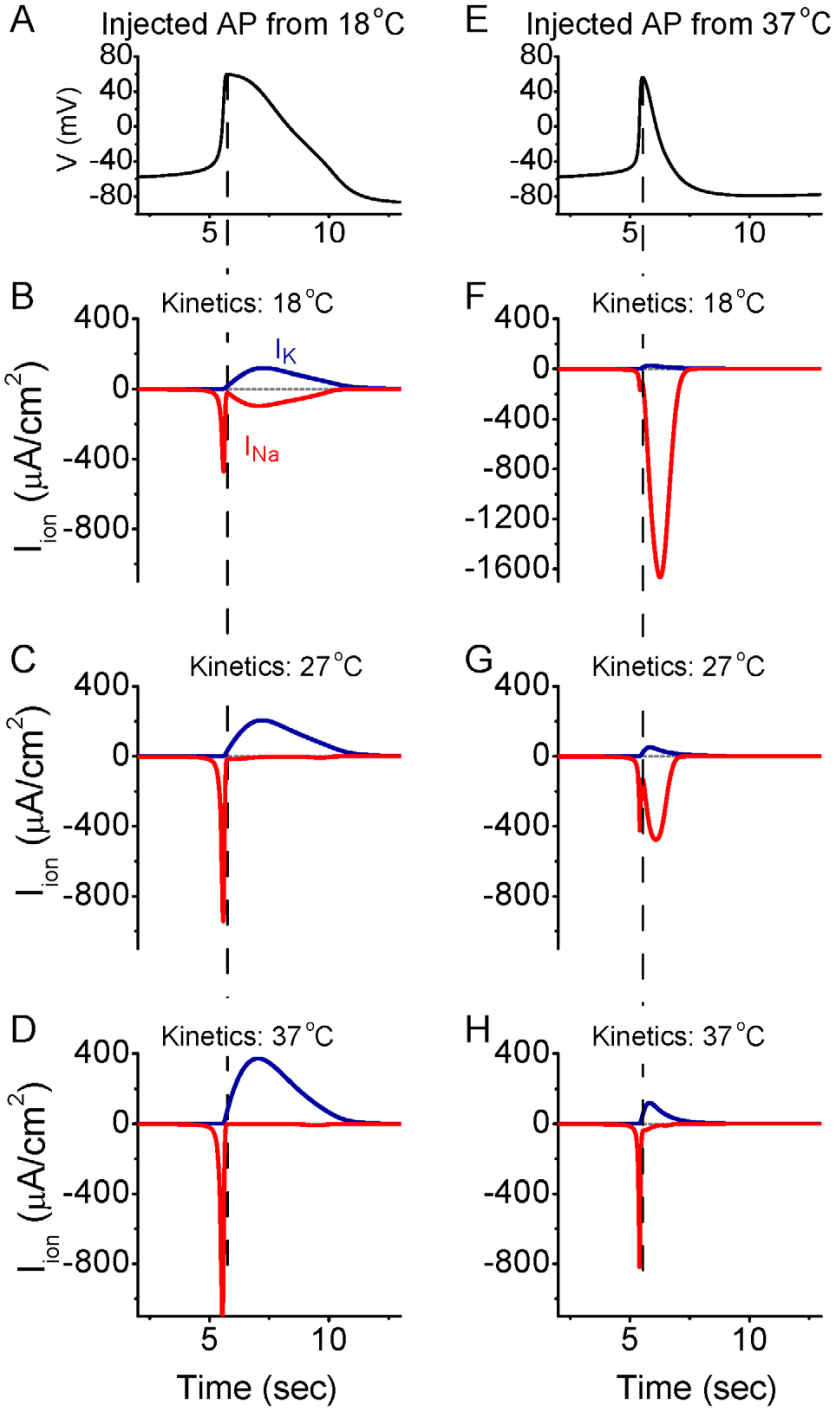
Changes in action potential shape do not explain the decrease in Na^+^ entry and Na^+^/K^+^ current overlap with increases in temperature. A. Action potential waveform occurring in the model axon at 18°C. B. Currents obtained from the model when the action potential in (A) was injected into the model, using ionic current kinetics obtained at 18°C. This result is the same as Figure 1B, 18°C. C–D. Running the model through the action potential waveform of (A), but with the channel kinetics of 27°C (C) or 37°C (D) results in a large decrease in Na^+^/K^+^ channel overlap (cf. B). E. Action potential waveform occurring in the model axon at 37°C. F. Injecting the action potential waveform in (E) into the model with Na^+^/K^+^ kinetics appropriate for 18°C results in a large inward Na^+^ current during the falling phase of the action potential, which overlaps extensively with the outward K^+^ current. G–H. Increasing the kinetics to those appropriate for 27°C (G) and 37°C (H) results in a large decrease in the overlap of Na^+^/K^+^ currents and the near disappearance of the Na^+^ current occurring during the falling phase of the spike (H). Dashed lines are aligned to the peak of the injected spike. See also supplemental figure S1.

Examining the relationship between action potential duration and excess Na^+^ entry ratio (inset in Figure 1D) may lead one to hypothesize that it is the shortening of the duration of the action potential that is the primary effect in the reduction of Na^+^ entry with each spike at higher temperatures (e.g. Figure 1A,B). To test this hypothesis, we fixed the action potential waveform to either that occurring in the cortical axon model at 18°C, or to that occurring in the model at 37°C (Figure 2). We then “injected” this waveform into the model and examined the amplitude –time course of the resulting Na^+^ and K^+^ currents, when their kinetics were set to temperatures varying from 6 to 37°C (Figure 2; supplemental Figure S1). When fixing the action potential waveform to that obtained at 18°C, we found that making the kinetics faster (i.e. raising temperature) dramatically reduced the overlap between the Na^+^ and K^+^ currents (Figure 2A–D) and reduced the excess Na^+^ entry ratio (supplemental Figure S1). Interestingly, fixing the action potential waveform at that occurring at 37°C, but using the ion channel kinetics occurring at 18°C, resulted in a large increase in Na^+^ entry and Na^+^/K^+^ current overlap, despite the fact that the action potential was much shorter in duration than that which normally occurs at 18°C (cf. Figure 2B, F). As with the long duration action potential (Figure 2A–D), making the kinetics of the underlying Na^+^/K^+^ currents faster (e.g. increasing temperature) while injecting the fixed 37°C action potential waveform, resulted in a marked decrease of the total Na^+^ current and overlap of Na^+^ and K^+^ currents (Figure 2E–H). Our analysis of these results reveals that the faster channel kinetics associated with increased temperature result in a marked decrease in the total Na^+^ current and Na^+^/K^+^ current overlap. This is primarily the result of an increase in Na^+^ channel inactivation, especially during the falling phase of the action potential, when the driving force on Na^+^ is especially large (see below). Thus, increasing temperature does not result in a decrease in total Na^+^ current or a decrease in Na^+^/K^+^ current overlap by decreasing the action potential duration. Rather, the increase in temperature results in both a decrease in spike duration as well as a decrease in excess Na^+^ entry owing largely to the increased rate of Na^+^ channel inactivation (see below).

**Figure 3:**
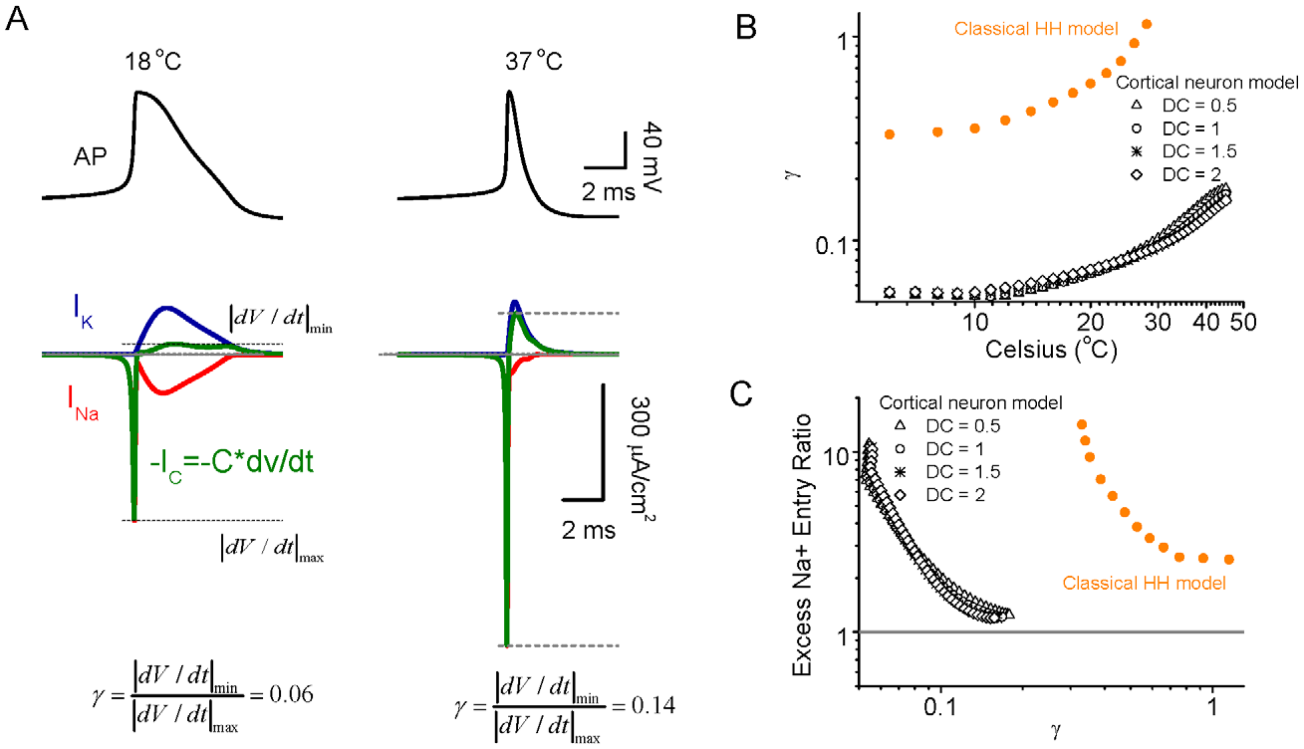
The ratio of absolute minimal dV/dt to maximal dV/dt is affected by the overlap level of Na^+^ and K^+^ currents. A. For a given action potential at 18°C, the Na^+^ and K^+^ currents have a large overlap, resulting in a cancelled effect in the total membrane charge current C*dV/dt and the ratio of absolute minimal dV/dt to maximal dV/dt ratio is small, approximately 0.06. For a given action potential at 37°C, the Na^+^ and K^+^ currents exhibit significantly less overlap, leading to a relatively large minimal dV/dt peak (similar in amplitude-time course as I_K_), while the maximal dV/dt peak resembles the amplitude-time course of I_Na_. The absolute minimal dV/dt to maximal dV/dt is increased to 0.14. B. The ratio γ (defined as the ratio of absolute minimal dV/dt to maximal dV/dt) increases as a function of temperature for both classical HH neuron and cortical neuronal models, owing largely to the decrease in overlap of Na^+^ and K^+^ currents with increases in temperature. C. The excess Na^+^ entry ratio decreases as a function of γ for both HH and cortical neuronal models.

Changes in the overlap of inward Na^+^ and outward K^+^ currents results in systematic changes in dV/dt of the action potential, and the ratio of the maximal falling to rising dV/dt values, with temperature (Figure 3). We define γ as (IdV/dtI_min_)/(IdV/dtI_max_). This variable is strongly influenced by the level of separation of the inward Na^+^ and outward K^+^ currents, and by other factors such as the peak amplitude of I_Na_ and I_K_. We include it here because it is a readily measureable variable in real neurons, and therefore useful for comparison with results of our model. Figure 3B shows that the value of γ increases nonlinearly with an increase in temperature, for both the classical Hodgkin-Huxley model as well as our simple model of a cortical action potential. Plotting the excess Na^+^ entry ratio as a function of γ revealed that as γ increases (representing in part decreased overlap of I_Na_ and I_K_), the excess Na^+^ entry ratio decreases (Figure 3C). The higher values of γ for the traditional HH model reveals a higher rate of spike repolarization (relative to spike depolarization) than is present in our simple cortical model.

**Figure 4:**
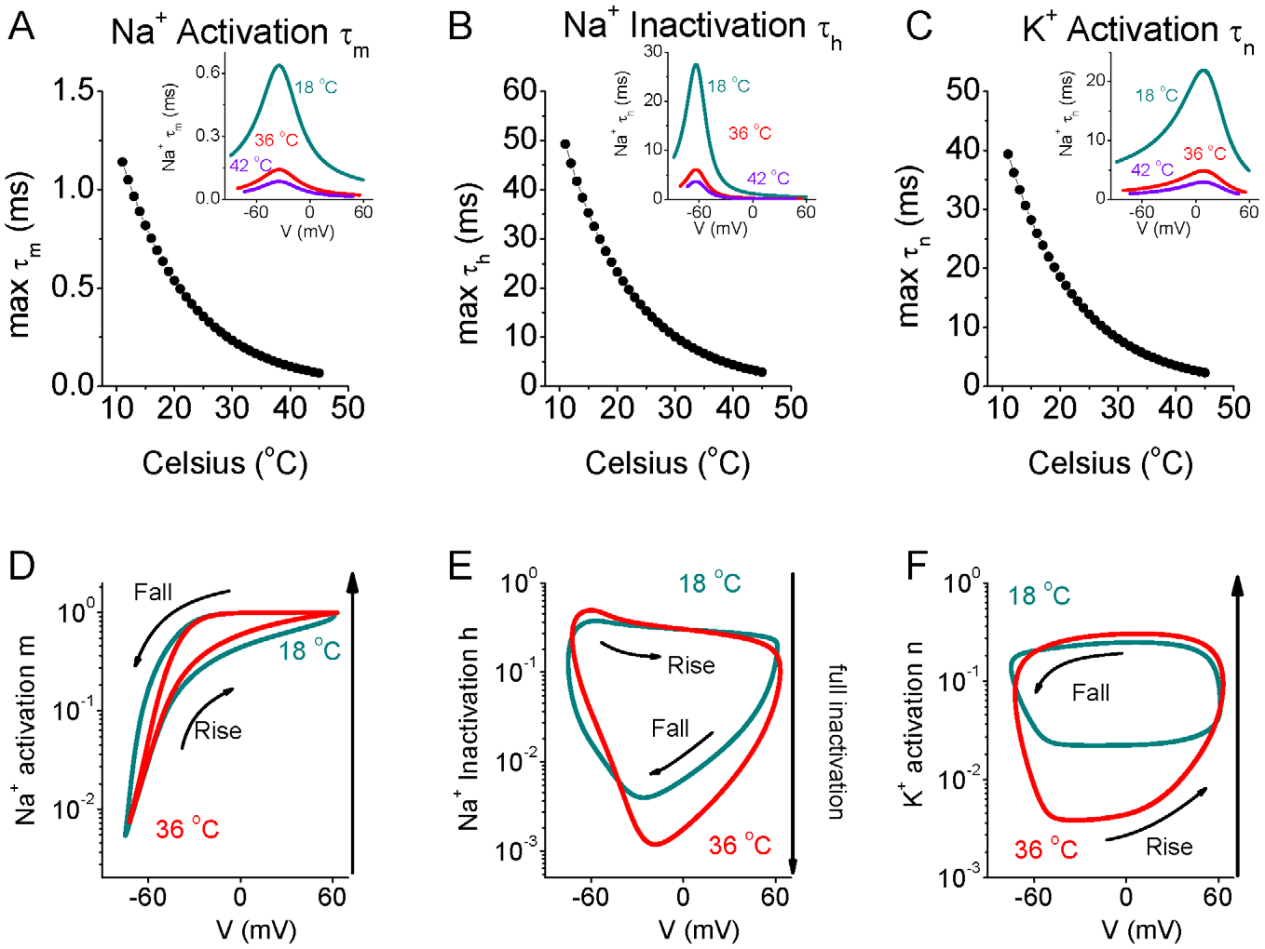
The kinetics of Na^+^ and K^+^ channels are heavily dependent on temperature change in the model neuron. A–C. The maximal value of Na^+^ activation time constant τ_m_ (A) and Na^+^ inactivation time constant τ_h_ (B), and K^+^ activation time constant τ_n_ (C) as a function of temperature. Inset: Time constants of the variables as a function of membrane potential for temperature T = 18°C, 36°C and 42°C, respectively. D–F. Phase plots of membrane potential V vs. Na^+^ activation variable m (D), Na^+^ inactivation variable h (E) and K^+^ activation variable n (F) for temperature T = 18°C, and 36°C, respectively. Note that at 36°C, the peak inactivation of the Na^+^ current is considerably more complete (e.g. attains a lower value) (E) and the K^+^ activation during the rising phase of the action potential is significantly less (F) than at 18°C.

We reasoned that the marked changes in overlap of inward Na^+^ and outward K^+^ currents during action potential generation with increases in temperature were due to changes in the kinetics of Na^+^ activation and inactivation, and K^+^ activation. Plots of the peak values of the time constants for activation of I_Na_ (τ_m_) and I_K_ (τ_n_) and inactivation (τ_h_) of I_Na_, revealed an exponential and strong decrease in all three with increases in temperature (Figure 4A–C). Phase plots of the I_Na_ activation (m) and inactivation (h) and I_K_ activation (n) variables versus membrane potential during generation of an action potential at 18 and 36°C revealed significant and important effects of temperature (Figure 4D–F). Since the currents vary over a very wide range of values, a logarithmic scale was used to monitor the smaller values during spike repolarization (Figure 4D–F). Interestingly increases in temperature from 18 to 36°C resulted in an increase in Na^+^ channel inactivation during nearly all phases of the action potential, with peak inactivation increasing from 0.5% of channels available at 18°C to only 0.1% available at 36°C (Figure 4E). In addition, increasing temperature from 18 to 36°C also results in a significant reduction in I_K_ activation during the rising phase of the action potential (Figure 4F). Even though increasing temperature increases ionic current kinetics substantially, the plot of m, h, and n versus membrane potential during action potential generation were substantially different from the steady state values of the currents (m_∞_; h_∞_, n_∞_; supplement Figure S2).

**Figure 5:**
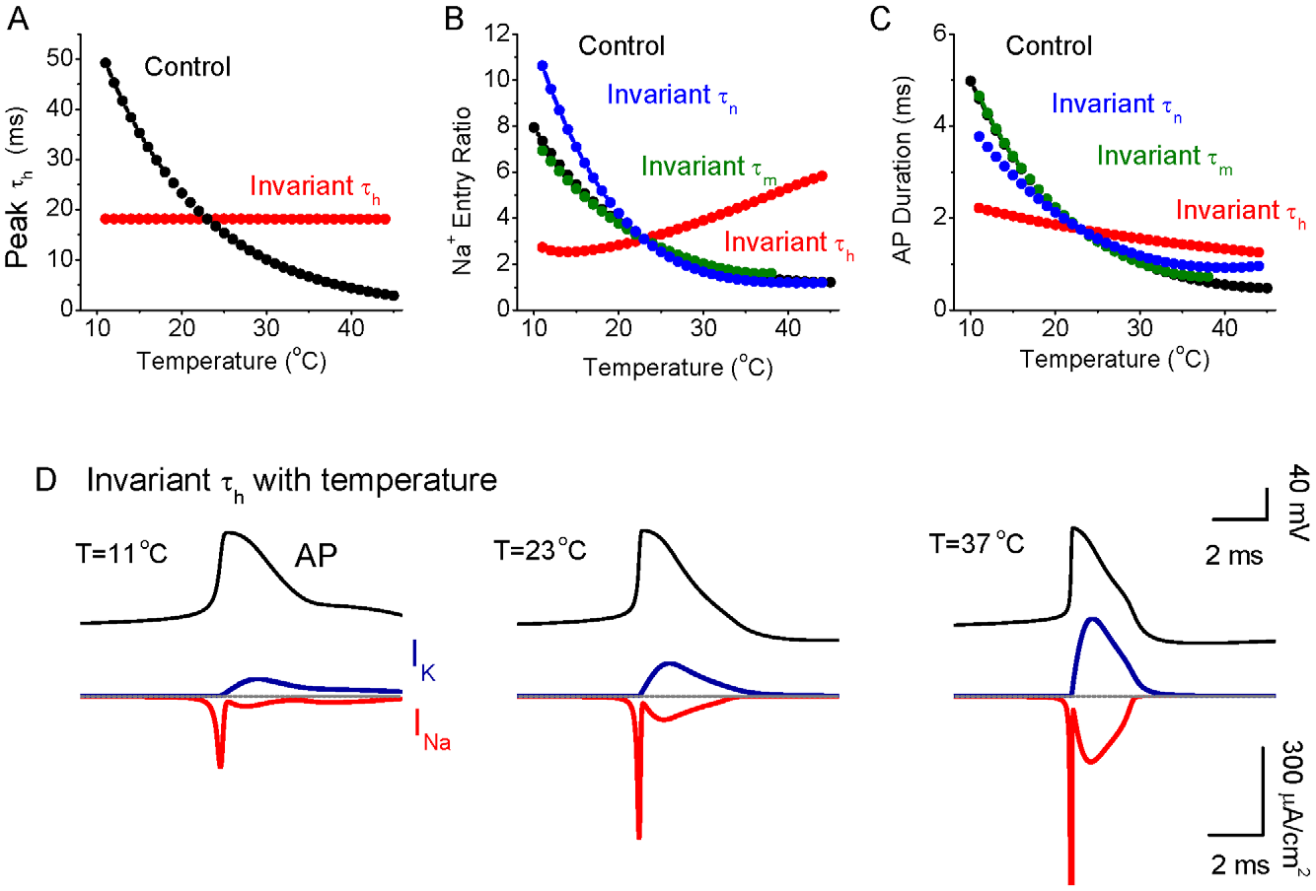
Contribution of changes in Na^+^ and K^+^ channel time constants on the effects of temperature on spike efficiency and duration. A. The maximum of Na^+^ inactivation time constant τ_h_ decreases as temperature increases for the normal model (control; black). For the test group, the maximum of Na^+^ inactivation time constant τ_h_ is invariant with temperature change (red). B. The Na^+^ entry ratio decreases as a function of temperature for control (black), while the Na^+^ entry ratio increases as a function of temperature when the inactivation time constant of I_Na_ (τ_h_) is fixed. Keeping I_Na_ activation time constant (τ_m_) invariant or I_K_ activation time constant (τ_n_) invariant with temperature reveals effects of temperature on Na^+^ excess entry similar to that in control. C. The half-height spike duration decreases as a function of temperature for the control group (black), while it becomes relatively independent to temperature change when I_Na_ inactivation time constant, τ_h_, is invariant. The test group with invariant τ_m_, or τ_n_, has similar behavior as that in control group. D. Example membrane potential and I_Na_, I_K_ for 11, 23, and 37°C when the time constant of I_Na_ inactivation (τ_h_) is kept constant. Note that the overlap of Na^+^ and K^+^ currents becomes larger with temperature, which is opposite to the normal situation (see Figure 1B).

**Figure 6:**
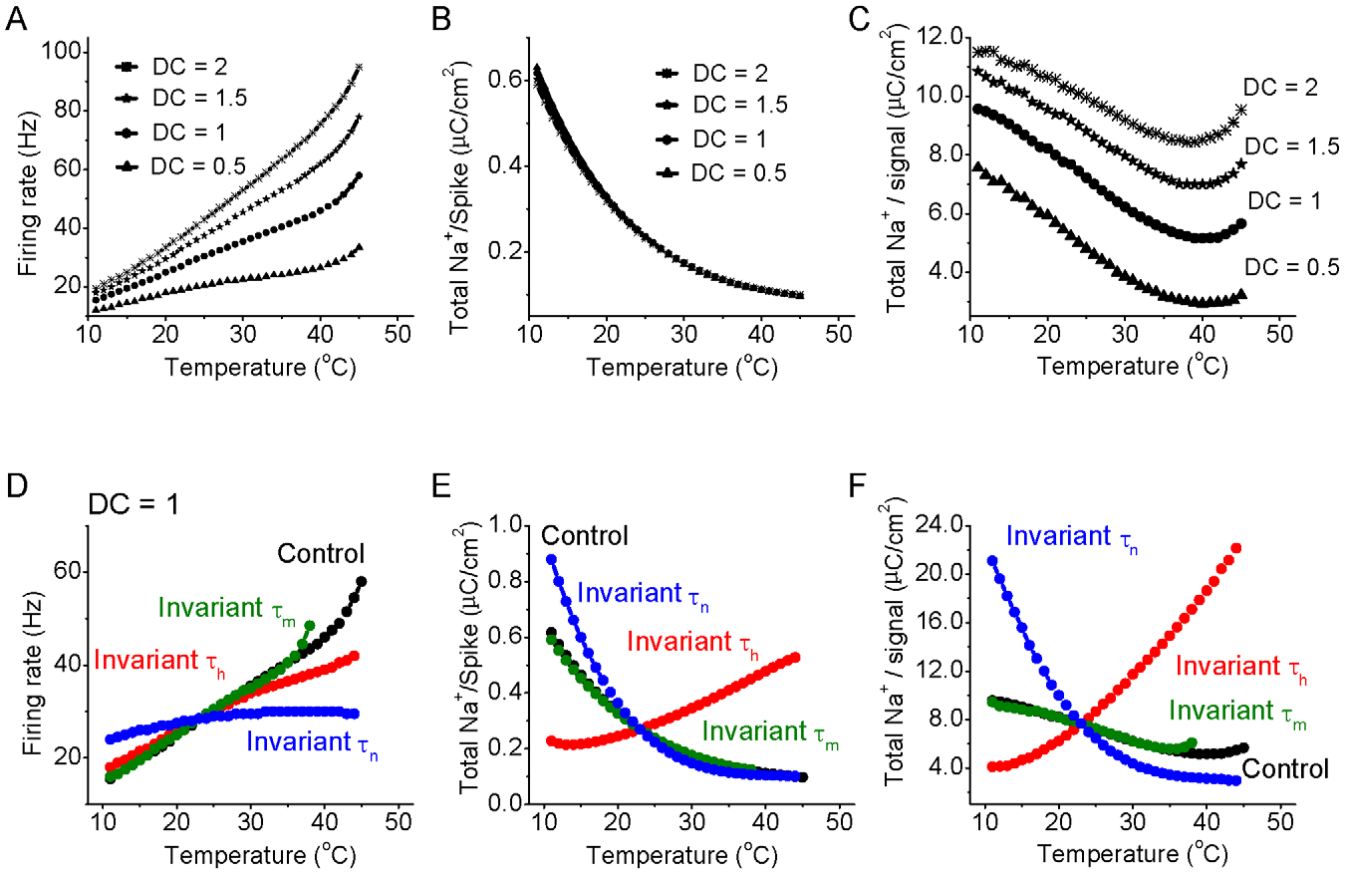
Effect of temperature on firing rate and total energy usage for the cortical model neuron in response to different intensities of DC input. A. The firing rate of the model neuron increases as a function of temperature for DC = 0.5, 1, 1.5 and 2×10^−2^ pA/µm^2^ (500 ms duration) respectively. B. The total sodium charge entering during one action potential decreases exponentially as a function of temperature for DC = 0.5, 1, 1.5 and 2×10^−2^ pA/µm^2^, respectively. C. For the conditions in the above four DC inputs, the total sodium charge (sodium charge per spike times the firing rate) for a given DC signal as a function of temperature. Note that the total Na^+^ entry reaches a minimum at a temperature of between 37–42°C. D. The firing rate increases as a function of temperature for the normal situation (black), and when τ_m_ (green), τ_h_ (red) and τ_n_(blue) are kept invariant. Notice that the I_K_ activation time constant τ_n_ is the key factor controlling the firing rate change as a function of temperature. E. For the above four situations, the total Na^+^ charge per single spike as a function of temperature. Notice that I_Na_ inactivation time constant τ_h_ is the key factor controlling the total Na^+^ charge per spike as a function of temperature. F. For the above four situations, the total Na^+^ charge per DC input (nC/cm^2^) as a function of temperature. Note that the total Na^+^ entry does not go through a global minimum for both test groups with temperature-independent τ_h_ and τ_n_.

Comparison of the results in Figures 1B and 4D–F suggest that the main contribution to the increase in efficiency of spike generation with increasing temperature is the strong increase in Na^+^ channel inactivation (Figure 4E), especially during the first half of spike repolarization, when Na^+^ activation is still high (Figure 4D), resulting in little excess Na^+^ entry during the falling phase of the action potential, as well as a decrease in spike duration. However, changes in I_K_ activation with increases in temperature may also contribute, by decreasing spike duration. These changes in I_Na_ activation/inactivation and I_K_ activation result in an exponential decrease in the total amount of Na^+^ that enters during each action potential (Figure 5B, black circles), thus decreasing the metabolic demand of spiking. The critical role of temperature dependent increases in the rate of I_Na_ inactivation was confirmed by keeping this rate constant (τ_h_) constant while allowing τ_m_ and τ_n_ to vary (Figure 5A, red circles). In this circumstance, the strong decrease in Na^+^ entry per action potential with increases in temperature was reversed, such that the Na^+^ entry ratio actually increased with temperature (Figures 5B, D, 6E). Keeping either I_Na_ activation rate (τ_m_) or I_K_ activation rate (τ_n_) constant individually did not abolish the strong decrease in Na^+^ entry/spike with temperature (Figures 5B, 6E). The decrease in spike duration with increase in temperature still occurred during constant τ_h_, τ_n_ or τ_m_, although this effect was greatly reduced when Na^+^ channel inactivation (τ_h_) was invariant (Figure 5C). This result indicates that the decreases in spike duration and excess Na^+^ entry ratio with temperature (effects that are inter-related; see discussion) result largely from changes in the kinetics of Na^+^ channel inactivation.

One complicating factor is that temperature affects the intrinsic excitability and spiking rate of neuronal elements [19], [21], [28], [29], [30], [31], [32], [33], [34]. Indeed, in our HH cortical model, increases in temperature result in an increase in firing rate in response to constant current pulses (0.005–0.02 pA/µm^2^, 500 ms). Interestingly, at higher temperatures (approximately 38–40°C), the firing rate in our models increases rapidly with increases in temperature (Figures 6A). Thus, for a constant current input, increases in temperature result in a marked decrease in the amount of Na^+^ that enters with each action potential (Figure 6B), but an increase in the number of action potentials generated in response to a constant input such as a square pulse of current. The total Na^+^ load (the number of spikes generated times the Na^+^ entry per spike) to a constant square pulse input decreases with temperature to a minimum at approximately 38–40°C. At temperatures above this minimum, the rapid increase in firing rate results in an increasing Na^+^ load on the neuronal process (Figure 6C).

**Figure 7:**
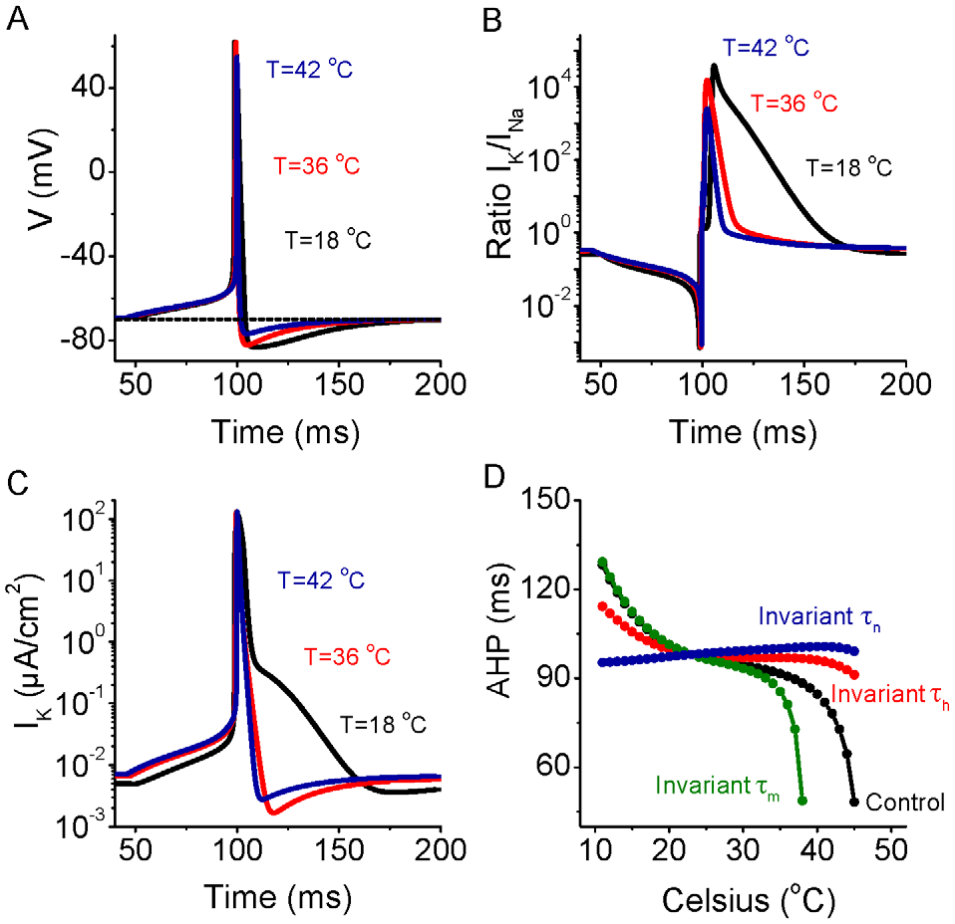
Increases in temperature result in a decrease in AHP duration. A. The action potential demonstrates a large and prolonged afterhyperpolarization (AHP) for low temperature (e.g., T = 18°C), and a smaller and shorter duration AHP for higher temperatures (e.g., T = 36, and 42°C, respectively). B. During an action potential, the ratio of potassium current I_K_ over I_Na_ as a function of time shows that there is a large amount of I_K_ available after the peak of action potential for low temperature (e.g., T = 18°C) in comparison with that at high temperatures (e.g., T = 36, and 42°C, respectively). C. Plot of the amplitude of I_K_ during action potential generation at different temperatures reveals that increasing temperature results in a marked reduction in the amplitude of I_K_, especially 20–60 msec after the spike, but also during the repolarizing phase of the action potential. D. Action potential AHP duration decreases slowly with increases in temperature for temperatures below approximately 37°C, while decreases rapidly for temperatures greater than approximately 37°C. Keeping the time constant of I_K_ activation (τ_n_) invariant abolishes this effect, while keeping the time constants of I_Na_ activation (τ_m_) or inactivation (τ_h_) invariant does not, although they do alter the magnitude and temperature range of the effect.

We suspected that this non-linear increase in firing rate with temperature in the simple HH model (Figure 6A) may result from a non-linear effect on spike afterhyperpolarization (AHP) since the amplitude and duration of the AHP largely determines neuronal discharge rate [31], [33] and it is known that increases in temperature decrease the duration of single spike afterhyperpolarizations in cortical neurons [31]. Plotting the duration of the model AHP (measured as the time to return to baseline following the generation of an action potential) as a function of temperature revealed a highly non-linear relationship, with AHP duration decreasing rapidly with increases in temperature above approximately 37°C (Figure 7A). Plotting the amplitude-time course of I_K_ (Figure 7C) as well as the ratio of the K^+^ current to Na^+^ current (Figure 7B) indicated that even though the K^+^ currents during the late phases of the AHP are small, lowering temperature results in a significant increase in their amplitude during the late phases of spike repolarization. At cold temperatures (e.g. 18°C), I_K_ became especially large even 25–60 msec after the spike. These changes resulted in a larger, more prolonged spike AHP (Figure 7). In confirmation of the important role of changes in I_K_ on these effects on the HH model, we found that keeping the activation rate (τ_n_) of I_K_ constant, while allowing the activation (τ_m_) and inactivate rates (τ_h_) of I_Na_ to vary with temperature, nearly abolished the ability of changes in temperature to cause non-linear changes in discharge rate (Figure 6A,D) as well as the non-linear decrease in AHP duration with increases in temperature (Figure 7D). Keeping either the activation rate (τ_m_) or inactivation rate (τ_h_) of I_Na_ constant, while allowing the other kinetic time constants to vary with temperature, did not change the presence of this non-linear relationship, although it did alter the range of temperatures over which it occurred (Figures 6A,D; 7D). As shown above, keeping the inactivation rate (τ_h_) of I_Na_ invariant inverted the relationship between the total Na^+^/spike and temperature, while keeping τ_m_ or τ_n_ invariant does not fundamentally alter this relationship (see Figure 6E). Consequently, the relationship between total Na^+^ entry per direct current pulse (Na^+^/signal) is strongly affected by keeping I_Na_ inactivate rate (τ_h_) invariant (Figure 6F). Keeping the activation rate of I_Na_ (τ_m_) constant has relatively little effect, while keeping the activation rate of I_K_ (τ_n_) constant exaggerates the decrease in total Na^+^/current pulse (Figure 6F).

**Figure 8:**
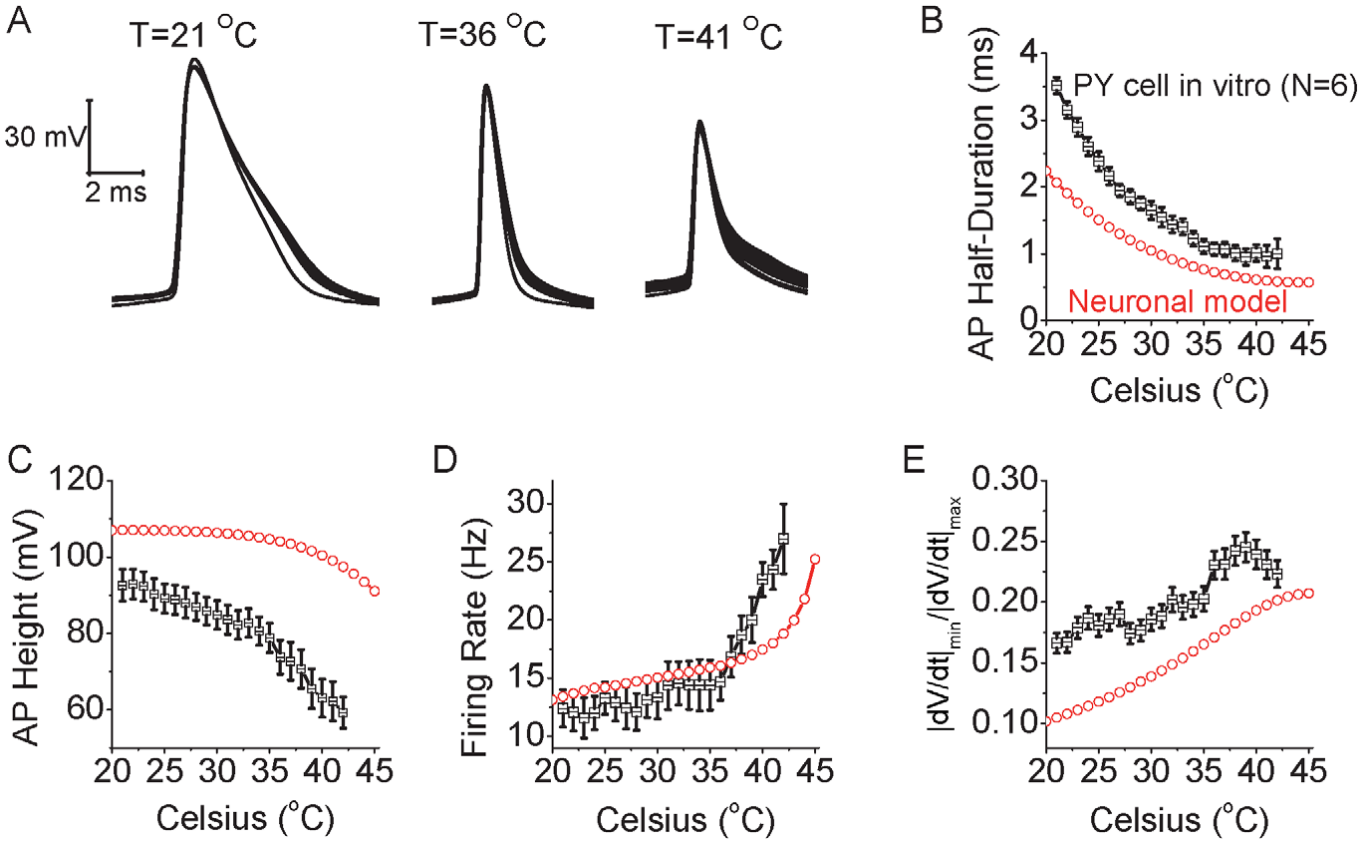
Increases in temperature increase the intrinsic excitability and change spike shape in pyramidal neurons as predicted by the HH model. A. Increasing temperature from 21°C to 36°C and 41°C results in shorter duration action potentials that are also decreased in amplitude in layer 5 pyramidal cells in vitro. Spikes were initiated with the intrasomatic injection of a depolarizing current pulse (100 pA, 500 msec duration). B. The spike duration, measure at half peak amplitude, decreases gradually as a function of temperature for pyramidal neurons (n = 6; black). The action potentials of the simulated neuron results show a similar property (red). C. Action potential amplitude decreases with increases in temperature for both pyramidal neurons in vitro (black) and for the model neuron (red). D. For a fixed current pulse amplitude, the average firing rate of pyramidal cells in vitro (n = 6) increases gradually as a function of temperature. Notice that for temperatures >36°C, the firing rate increases more rapidly. The model neuron (red) also exhibits a non-linear increase in firing rate to a current pulse with increases in temperature. Differences between the real neuron and model results presumably arise from the complex morphology and properties of ionic channels not included in the simple HH model. E. Same data set as B, the average value of dv/dt ratio (minimum dV/dt divided by maximum dV/dt during the spike) increases gradually as a function of temperature for the recorded pyramidal cells in vitro (n = 6) (black). The model neuron exhibits a similar relationship (red).

**Figure 9:**
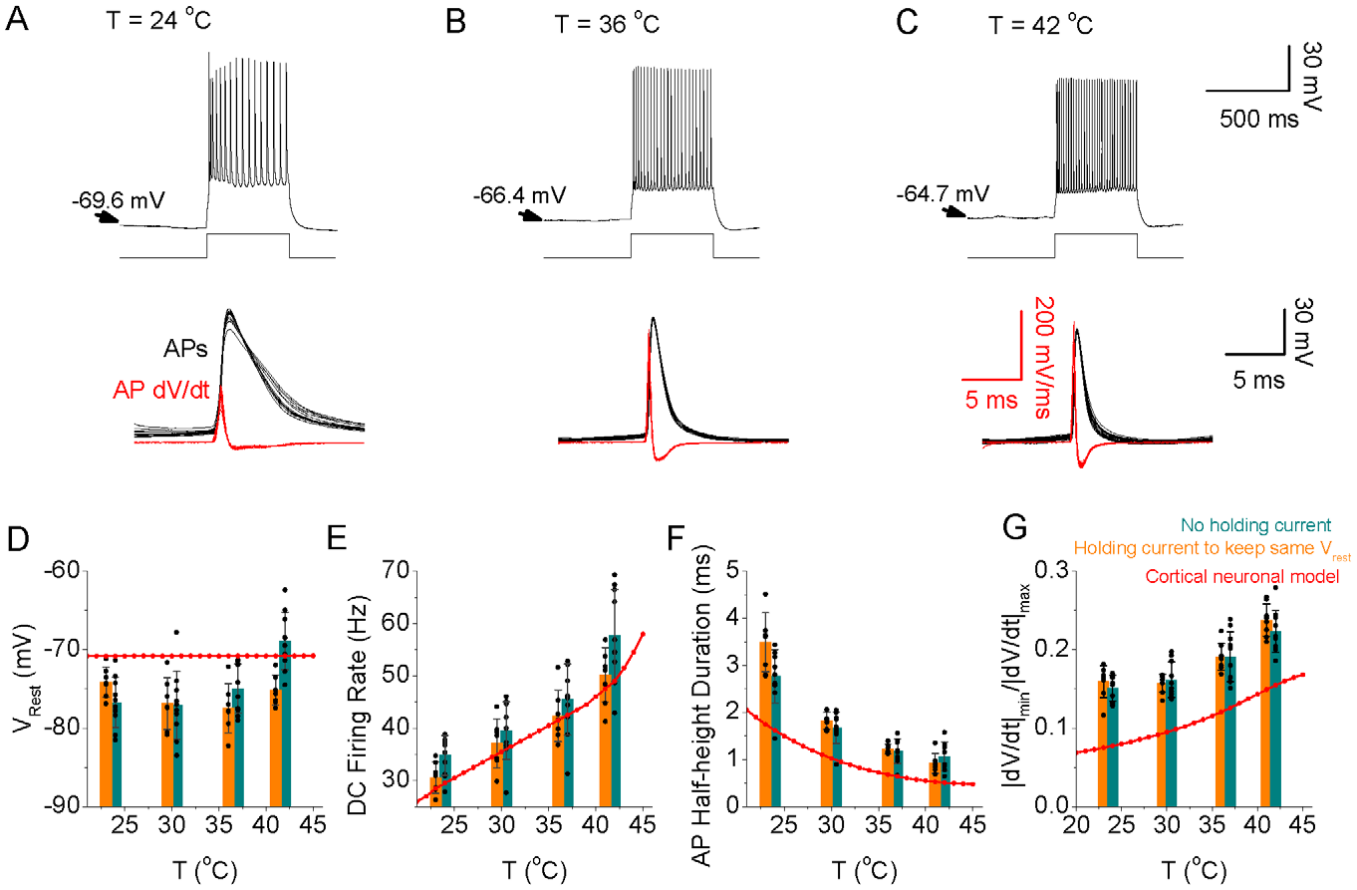
Increasing temperature increases neuronal responsiveness in layer 2/3 entorhinal cortical pyramidal cells. Intracellular recordings from a layer 2/3 pyramidal neuron to a DC input (150 pA, 500 ms duration) at a temperature of 24, 36, and 42°C respectively (A–C). Note that the neuron not only increases its firing rate, but also decrease its spike duration, to the current pulse with an increase in temperature. The dV/dt of action potentials shows a gradual increase of the peak rate of rise and fall with increases in temperature. D–G. Bar graphs illustrating that increasing temperature results in a depolarization of the membrane potential (cyan bars), an increase in spike rate, a decrease in single spike duration, an increase in the ratio of the absolute minimal dV/dt to maximal dV/dt, respectively. Similar effects were obtained when the depolarization of the membrane potential that was particularly prominent at 42°C was kept relatively negated by adjusting the holding current (orange bars). As a comparison, the cortical model results for a DC input (1×10^−2^ pA/µm^2^, 500 ms duration) are represented by the red line.

Our HH-style simulation results suggest that increases in temperature may result in several important changes in neuronal action potential generation: 1) action potentials will become shorter in duration and smaller in amplitude, with a marked decrease in overlap of the inward Na^+^ and outward K^+^ currents, resulting in a marked reduction in Na^+^ load/spike; 2) The firing rate of the neuronal process to a constant increases will increase non-linearly with changes in temperature, particularly at temperatures above approximately 37°C. Next we tested whether or not these predictions would be confirmed in somatosensory layer 5 and entorhinal layer 2/3 cortical pyramidal cells recorded in vitro in response to constant current pulse injection (Figures 8,9), or during the spontaneous generation of the cortical slow oscillation (supplemental Figure S3).

As predicted by HH simulations, increases in temperature resulted in a significant decrease in spike duration (Figure 8A, B), spike height (Figure 8C), and a steady increase in firing rate in layer 5 somatosensory cortical pyramidal cells between approximately 20 and 35°C in response to the intrasomatic injection of a constant current pulse (500 msec, 100 pA; Figure 8D; n = 6 cells). At temperatures above approximately 35°C, the firing rate increased markedly to the constant current pulse, such that the slope of the frequency-temperature (f-T) relationship increased dramatically (Figure 8D). Interestingly, in layer 2/3 cortical pyramidal neurons spontaneously generating the recurrent network-driven slow oscillation [35], increases in temperature also resulted in a marked increase in neuronal spiking during Up states (supplemental figure S3; n = 10 cells), as reported previously [36]. Similarly, layer 2/3 cortical pyramidal cells also increased their responsiveness to the intracellular injection of a current pulse (150 pA; 500 msec) with increases in temperature from 23 to 42°C (Figure 9A–C, E; n = 10 cells). Increases in temperature resulted in a small depolarization of layer 2/3 entorhinal cortical pyramidal cells (−76.7+/−3.2 mV 23°C; −74.9+/−3.1 mV 36°C; −68.9+/−3.7 mV 42°C; p<0.01, t-test between Vm at 23 and 42°C) (Figure 9D). The increase in responsiveness to a constant current pulse was only partially due to the small depolarization of the resting membrane potential with increases in temperature. Compensation for the change in membrane potential with the intracellular injection of current did not abolish the increase in responsiveness with temperature (Figure 9E, compare orange and green bars; n = 10). Since the number of action potentials and the discharge rate changed with temperature, we were unable to measure the effects of temperature on single spike or spike train induced afterhyperpolarizations. Previous results have demonstrated that decreases in temperature slow the kinetics of fast, medium, and slow afterhyperpolarizations, although to a differential degree, presumably owing to the properties of intracellular Ca^2+^ signaling [31].

In both layer 5 and layer 2/3 pyramidal cells, as predicted by the HH model, the action potential duration (as measured at half amplitude) decreased exponentially with increases in temperature (Figures 8B, 9F), action potential amplitude decreased with temperature (Figures 8A, B; 9A–C), and the ratio of the minimum dV/dt to maximum dV/dt during the spike increased with temperature (Figures 8E, 9G). These results confirm the validity of this model as a basic representation of the effects of temperature on cortical action potential generation. Increases in temperature from 23 to 42°C also resulted in a significant decrease in apparent input resistance in both layer 5 (105+/−15 MOhms 23°C; 88+/−11 MOhms 42°C; p<0.01) and layer 2/3 (246+/−25 MOhms 23°C; 164+/−31 MOhms 42°C; p<0.01) pyramidal neurons. Increases in temperature from 23 to 37°C also resulted in a small decrease (−1.6+/−1.7 mV; n = 10; p<0.05) in spike threshold for layer 2/3 pyramidal neurons (−47.2+/−3.4 mV 23°C; −48.8+/−2.9 mV at 37°C). Simulation of changes in apparent input resistance and resting membrane potential reveal that, as expected, both decreasing input resistance and hyperpolarization decrease firing rate at nearly all temperatures, without changing strongly the shape of the f-T relationship (supplemental Figure S4A) or the relationship between temperature and total Na^+^ entry per current pulse (supplemental Figure S4B). These results suggest that decreases in apparent input resistance with temperature will at least partially offset the depolarization of resting membrane potential (Figure 9D). Simulations also indicate that decreases in apparent input resistance and membrane time constant with increases in temperature will result in only small increases in excess Na^+^ entry per action potential (see supplement Figure S5), although these may increase the energetic costs of the network as a whole, owing to the requirement for more action potentials per unit of time in the presynaptic neuronal network in order to reach firing threshold in the postsynaptic cell.

**Figure 10:**
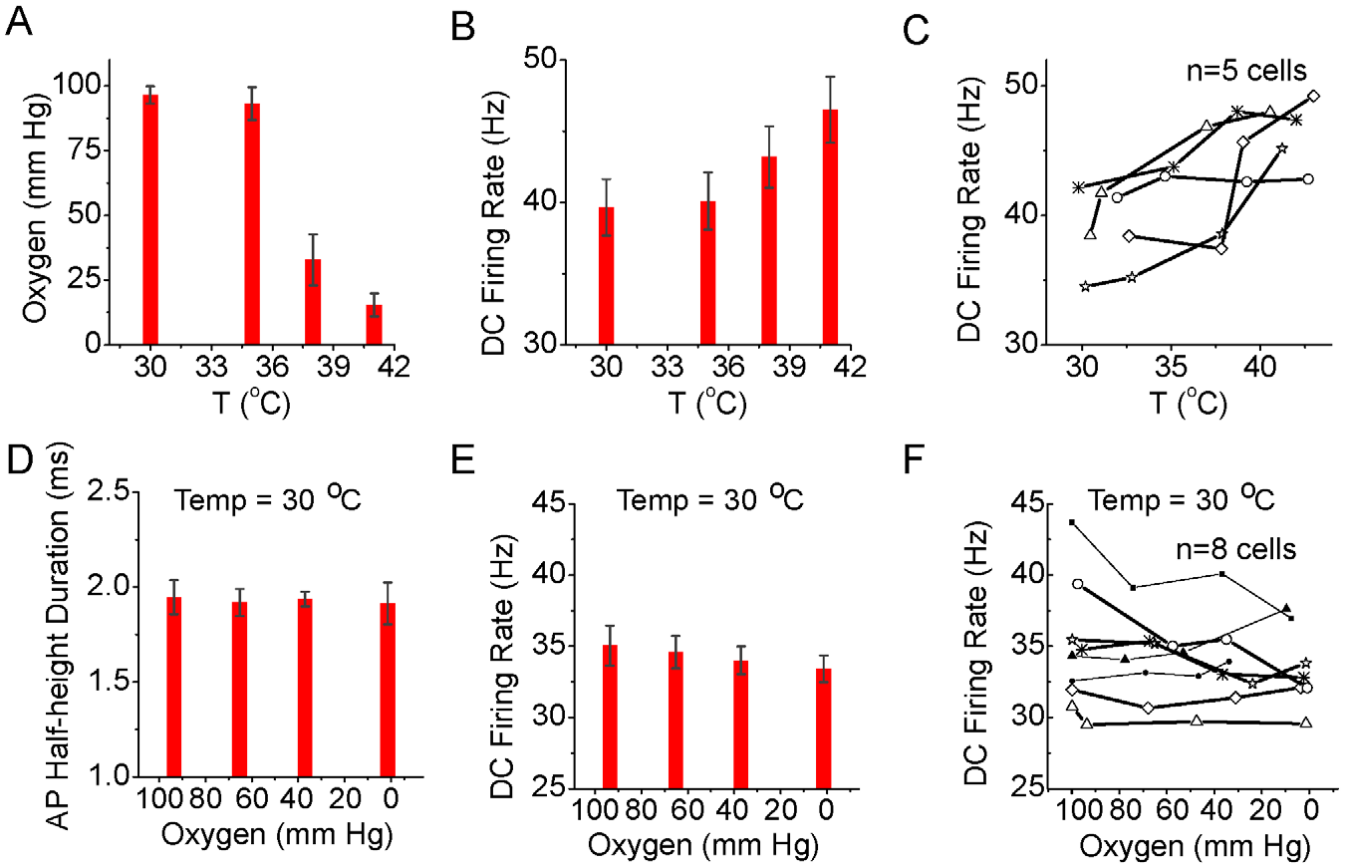
Increasing temperature decreases oxygen concentration and decreasing oxygen content decreases neuronal excitability. A. Oxygen levels (mm Hg), as measured at the upper surface of the slice, at different temperatures. Increasing temperature can dramatically decrease oxygen levels. B. Response of layer 2/3 pyramidal cells (n = 5) to the intracellular injection of a depolarizing current pulse (200 pA, 500 ms) at different temperatures (same experiments as in A). C. Plot of response of individual cells to the current pulse at different temperatures. D–F. Decreasing oxygen levels (mm Hg) at a constant temperature of 30°C does not have a significant effect on the duration of action potentials (D), but does decrease average firing rate (E), as seen in individual recordings (F; n = 8 cells).

One possible confounding factor in our in vitro recordings is that increasing temperature indirectly increased neuronal excitability through decreasing the oxygen content of the bathing solution. To examine this possibility, we measured the oxygen content of the ACSF at the upper interface of layer 2/3 of the entorhinal cortical slice and the bath solution while varying temperature (Figure 10A). We then independently reduced the oxygen content of the ACSF while maintaining a constant temperature (Figure 10; n = 8). Increasing temperature from 30 to 41°C resulted in a marked decrease in oxygen content of the bathing solution, from an average of 96.5 (+/−3.4; n = 5) to 15.4 (+/−4.5) mm Hg (Figure 10A; n = 5). As observed previously, increasing temperature resulted in an increase in pyramidal cell action potential response rate to a constant current pulse (Figure 10B, C). In contrast to these effects, acutely decreasing ACSF oxygen content from 98.5 (+/−3.2) down to 6.4 (+/−4.3) mm Hg did not significantly affect action potential duration (Figure 9D), and resulted in a small, but statistically significant (p<0.01; t-test between lowest and highest mm Hg) decrease in action potential response rate (from 35.1+/1.4 Hz at 98.5 mm Hg to 33.4+/−0.95 Hz at 6.4 mm Hg) to the intracellular injection of a depolarizing current pulse (Figure 10E, F). These results indicate that decreases in ACSF oxygen content do not explain the increase in neuronal excitability associated with increases in temperature, and if anything, result in the under-estimation of the magnitude of this effect.

## Discussion

Increases in temperature have marked and strong effects on action potential generation, and these in turn impact upon the energy efficiency of neuronal activity. Here we demonstrate that increases in temperature result in a large increase in the energy efficiency of single action potential generation owing to the increased rate of Na^+^ channel inactivation and subsequent decreased spike amplitude, duration and overlap between inward Na^+^ and outward K^+^ currents. Thus, increases in temperature naturally result in a marked decrease in excess Na^+^ entry during spike generation, reducing the need for activation of the Na^+^/K^+^ ion pump (ATPase), and thus reducing energy expenditure. This result suggests that the higher body temperature of endotherms such as mammals (versus ectotherms such as squid) has the advantage of resulting in a marked increase in energy efficiency of single action potential generation. However, increases in temperature also resulted in an increase in spike discharge rate to a constant amplitude input or during spontaneous network activity, owing in part to decreases in the amplitude and duration of K^+^ currents initiated by action potentials [31], [33]. Interestingly, Hodgkin-Huxley style models, and whole cell recordings from cortical pyramidal cells, reveal increases in firing rate to be particularly pronounced at temperatures above approximately 37°C. Thus, even though individual spike efficiency increases with increasing temperature, the enhanced firing rate raises energy requirements. Maximal overall energy efficiency in neuronal responsiveness is observed near 37°C.

Energy expenditure in the brain is divided among requirements for action potentials, synaptic potentials, maintenance of resting membrane potential, axonal and dendritic transport, and other metabolic functions [1], [5], [6], [7], [10], [11], [21], [37]. Estimates of the relative contribution of energy demands related to action potential generation to the overall energy needs of the mammalian brain have varied from approximately 25 to more than 50% [5], [10]. The estimates of high energy demands related to action potentials have led to the speculation that average firing rates in the brain may be very low (<0.2 Hz) [6], a hypothesis that has some experimental support [38]. However, the estimates of unusually high energy demands of action potential generation have been based largely upon the observation by Hodgkin [9] of four times excess Na^+^ entry during spike generation in the squid giant axon [5], [13]. This observation and calculation was performed at 18°C, and the results must be corrected to 36–39°C in order to be applied to the mammalian brain. Unfortunately, this correction has not been systematically applied. Our calculations predict that at 37°C, there should be relatively little excess Na^+^ entry during action potential generation(excess ratio of around 1.3) (Figure 1A). Recent observations in cortical pyramidal cells and axons at 37°C confirm the high energy efficiency of action potential generation in these neurons, owing in part to a markedly reduced overlap in inward Na^+^ and outward K^+^ currents [12]. Taking these observations into account suggests that the energy load of action potential generation in endothermic animals may be as much as three times lower than previously calculated, allowing for a significantly higher average discharge rate. Increases in temperature increase the kinetics of conformational state changes in all ionic channel types involved in action potential generation, with a Q_10_ ranging from 1.5 to 4 [17], [18], [26], [27]. Particularly important for action potential energy efficiency is the temperature dependent increase in rate of Na^+^ channel inactivation [13], [18], which markedly reduces the duration of action potentials, with a smaller effect on action potential amplitude, as well as decreasing the overlap between inward Na^+^ and outward K^+^ currents owing to nearly complete Na^+^ channel inactivation during the falling phase of the spike at 37°C (Figures 1–3).

Normal mammalian brain temperature varies from approximately 36–39°C, depending upon state of the animal (e.g. resting, exercise) and location within the brain, although some mammals (and some species of birds) can exhibit brain temperatures as high as 45°C [19], [20], [21], [22], [23], [24]. Under stress, such as during infection or environmental conditions that reduce the effectiveness of body cooling mechanisms (e.g. high humidity and temperature), human brain temperature can reach levels in excess of 40°C [21]. Rapid rises in temperature to high levels, especially in children and adolescents, can result in the initiation of a febrile seizure [39], suggesting that the operational balance of excitation and inhibition is temperature dependent. Even small changes (e.g. 1–2°C) in brain temperature can have significant effects on network function. Prior investigations of the effect of increased temperature on neurons reveal consistent changes in action potentials including decreased duration and amplitude, increased rate of rise and fall, and decreased spike afterhyperpolarization, as partially predicted by HH equations for changes in the kinetics of the underlying ionic channels [19], [21], [28], [29], [30], [31], [32], [33], [34]. In addition, increases in temperature typically result in a decrease in membrane input resistance, and can, in some cell types, depolarize the membrane potential through the activation of TRPV channels which conduct cations and have a reversal potential well above rest [32], [40], [41], [42]. Interestingly, even without changes in membrane potential, simple HH equations predict that increases in temperature will result in enhanced neuronal responses to a constant current input, owing to increases in discharge frequency. This effect of temperature results largely from strong temperature dependent decreases in the spike afterhyperpolarization owing to decreased amplitude and duration of outward K^+^ currents activated by action potentials. These decreases in K^+^ current amplitude and duration may result from decreases in activation owing to the shortened and smaller action potential (which would allow, for example, less Ca^2+^ to enter per action potential), changes in the kinetics of second messenger events within the neuron following the action potential, or changes in the kinetics of the K^+^ channels themselves [31]. In our recordings, the firing rate of cortical pyramidal cells to a constant input was particularly enhanced at temperatures above approximately 36°C (Figure 6A, 8D,9E). Although this increase in firing rate was similar to that predicted by a simple HH model, it occurred at a lower temperature than predicted (Figure 8D), presumably owing to the presence of a wide variety of complex ionic currents, such as Ca^2+^ activated K^+^ currents, in real neurons that were not included in our simple simulation [16], [31].

Different neuronal subtypes in the brain vary in their energy efficiency of action potential generation owing in part to differences in overlap of action potential-related Na^+^ and K^+^ currents [15], [43]. In a subtype of cortical interneuron, the fast spiking cell, as well as cerebellar Purkinje neurons, the generation of short duration action potentials which have a short relative refractory period extends the dynamic range of the neuron, allowing for the generation of actions potentials from low to high (hundreds of Hz) frequencies. The short duration of these action potentials is achieved through the presence of rapidly activating K^+^ currents responsible for spike repolarization [44]. However, this mechanism of short spike generation, as opposed to rapid Na^+^ channel inactivation, significantly increases the energy demands on the neuron, owing to significant overlap of the inward Na^+^ and outward K^+^ currents [14], [43]. A subset of cortical pyramidal cells also exhibit unusually short duration action potentials, owing to rapid action potential repolarization [45]. Presumably these short duration action potentials are generated at the cost of energy efficiency. Short duration action potentials, however, do not always imply a high energetic cost. The action potentials generated in the axon of cortical pyramidal neurons are shorter in duration than those in the soma, although the axonal spikes exhibit relatively little overlap in Na^+^ and K^+^ currents, resulting in high energy efficiency [12], [13]. Dual somatic/axonal recordings from these neurons demonstrate a marked difference in K^+^ channel properties between the soma and axon, with Kv1.1/1.2 channels being prevalent in the axon initial segment [46], [47]. In addition, somatic and axonal recordings from dentate granule cells reveal that the kinetics of activation and inactivation of voltage-dependent Na^+^ channels in the axon initial segment are approximately 2X faster than those in the soma [13]. This rapid inactivation kinetics of axonal Na^+^ channels may result from the presence of 1–3 auxiliary subunits in axonal locations [48], [49], [50]. Thus, while temperature has a large effect on energy efficiency of action potential generation, alterations in types and properties of the ionic channels underlying the generation of spikes also contributes significantly to inter- and intra-cellular variations. Indeed, it is well known that animals can alter their neuronal and action potential properties to adapt to varying environmental temperatures [51], although it is not yet known how these changes affect the energy efficiency of spiking.

Sparse neuronal discharge, in which the impact of each action potential is maximized in the task of the network in which it is embedded, is a powerful means to reduce overall energy expenditure [38]. In behaving animals, different types of cortical neurons, varying by cell type and layer, exhibit differing levels of neuronal activity, from very sparse (e.g. layer 2/3 and 6 pyramidal cells), to moderate (e.g. layer 5 pyramidal cells) to highly active (e.g. fast spiking inhibitory interneurons) [52], [53]. We presume that the electrophysiological properties of each neuronal subtype (and even sub-portions of each neuron) are adjusted to optimize their unique roles in the network, while simultaneously working within the limits of energy availability and capacity to dissipate heat.

Our observation that total Na^+^ load (spike rate×Na^+^/spike) on a neuron reached its minimum at about 37°C suggests that neurons may have optimized their energy use for this temperature range, as has been observed for a number of cellular and organ functions such as enzymatic function and the balance of heat production/loss in an ambient external environment of 25°C [21]. Our results demonstrate that there is no need to hypothesize highly specialized changes in the mechanisms of action potential generation in the evolution from invertebrates to mammals, or from poikliotherms to homeotherms – simply the increase in body temperature, an energy expensive commodity, results in a large and significant decrease in the cost of spike generation and propagation, thus allowing for larger and more complex brains.

## Methods

Experiments were carried out in accordance with all relevant NIH and institutional guidelines on the proper care and use of animals in research. Experiments were performed on slices of mouse somatosensory (20–45 days old) or entorhinal (12–21 days old) cortex maintained in vitro in a submerged style recording chamber. The ACSF contained (in mM): NaCl 126, KCl 2.5, MgSO_4_ 2, CaCl_2_, 2, NaHCO_3_ 26, NaH_2_PO_4_ 1.25, dextrose 25 (315 mOsm, pH 7.4). For maintaining the slow oscillation in vitro, the extracellular [Ca^2+^] and [Mg^2+^] were reduced to 1.0 mM [35]. Recordings were done on an upright infrared-differential interference contrast (IR-DIC) microscope (Zeiss Axioskop 2 FS plus). Cortical slices were suspended on a net, oxygenated solution flowed over both the upper and lower surfaces, at a rate of 3–5 ml/min. The membrane potential in our whole cell recordings was corrected for Donnan liquid junction potentials of 10 mV [54], [55]. Temperature was regulated through a Warner Instruments Corporation two channel temperature regulator (Model TC344B) which controls not only the temperature of the ACSF as it enters the recording chamber, but also the temperature of the stage upon which the chamber is mounted. The temperature of the ACSF was monitored by a thermister placed at the outflow of the solution, approximately 1–2 mm from the cortical slice.

Whole-cell recordings were achieved from the soma using a Multiclamp 700B amplifier (Axon Instruments, Union City, CA) as described previously [47]. Pipettes had an impedance of 5–6 MΩ and were filled with an intracellular solution that contained (in mM): KGluconate 140, KCl 3, MgCl_2_ 2, Na_2_ATP 2, HEPES 10, EGTA 0.2 mM, pH 7.2 with KOH (288 mOsm). All statistical comparisons were performed using a two-tailed t-test. All results are reported as mean +/− standard deviation, and illustrated as mean +/− standard error of the mean.

### Methods for Computational Model of Action Potential Generation in Cortical Neurons

1. **Original Hodgkin-Huxley Model.** The classical Hodgkin-Huxley equation describing the membrane potential as a function of all the currents that flow across the axon membrane, is
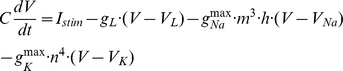
where V is the voltage difference across the membrane (in mV), C = 1 uF/cm^2^ is the specific membrane capacitance, I_stim_ is the input stimulus, g_L_ = 0.3 mS/cm^2^, g_Na_^max^ = 120 mS/cm^2^, g_K_^max^ = 36 mS/cm^2^, are the leak, maximal sodium and maximal potassium conductance per unit membrane area, respectively. V_L_ = −54.4 mV, V_Na_ = 50 mV, and V_K_ = −77 mV are reversal potentials of leak, sodium and potassium channels, respectively. The gate variables m, h, and n are dimensionless activation and inactivation variables, which describe the activation and inactivation processes of the sodium and potassium channels, each of which is governed by the following differential equations:
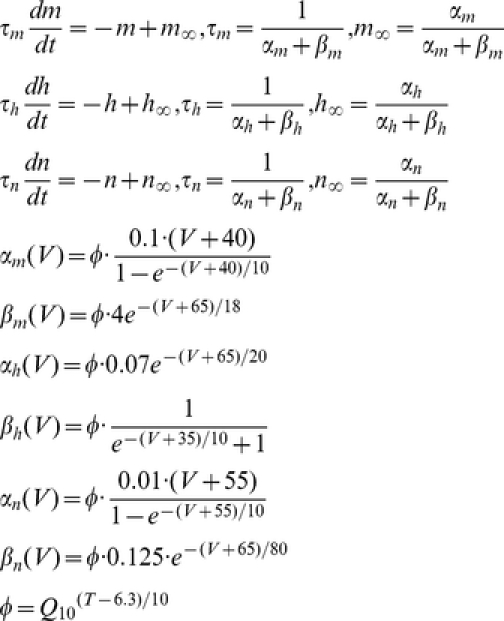
where *Φ* regulating the temperature dependence, known as the Law of Arrhenius, with Q_10_ = 3. The sensitivity of the Na^+^ current kinetics to temperature has been studied in a variety of preparations, where the Q_10_ for inactivation is, on average, 2.95 and the Q_10_ for activation is 2.68 (see Table 2; [18]).
2. **Hodgkin-Huxley-Style Cortical Neuronal Model.** To have a comparison with the results from the original HH model, and also to address the key factors contributing to energy efficient action potentials, only three major ionic voltage-dependent currents have been used in our cortical model: fast Na^+^, I_Na_, fast K^+^, I_K_, and a leak current, I_L_. The equations describing the voltage and time dependence of the Na^+^ and K^+^ conductances were based upon previous publications [56], whose channel kinetics are modified based on models of cortical neurons [25], [57], [58] and experimental studies [13], [25], [59], [60]. The equations describing the cortical axon single compartment model:
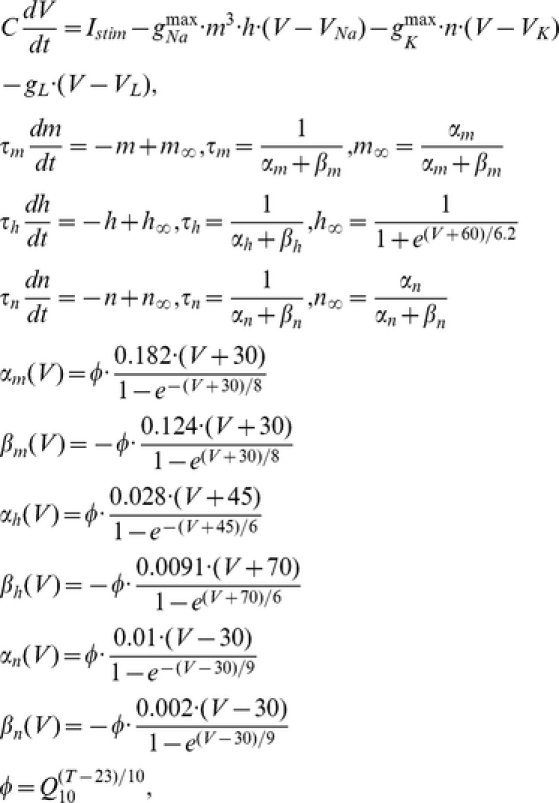
where the Q_10_ effect, described by *Φ*, regulates the temperature dependence of the rate constants, with Q_10_ = 2.3 [26], [27]. The relationship between temperature and I_Na_ and I_K_ activation and inactivation is not monotonic and varies in different species [17]. The reversal potential for Na^+^ and K^+^ currents was adjusted according to the Nernst equation with each change in temperature. Similar results were obtained with a variety of values for Q_10_. Using a Q_10_ of 3, for example, yielded similar results in spike efficiency and changes in spike rate with temperature. In our cortical model, we slightly adjusted Na^+^ kinetics to be faster than what we have used previously [25], owing to recent experimental observations by Schmidt-Hieber and Bischofberger [13]. The parameters used in our present cortical neuronal model are: membrane capacitance = 0.75 µF/cm^2^, g_Na_ = 1500 pS/µm^2^ (based on recent experimental results [13], [59], [61], [62], [63], density of g_K_ = 400 pS/µm^2^ [60] and gleak = 0.33 pS/µm^2^. The reversal potentials are V_L_ = −70 mV, V_Na_ = 60 mV, and V_K_ = −90 mV for leak, sodium and potassium channels, respectively. The injected current used in the model of action potential generation was 0.5×10^−2^ pA/µm^2^ for Figure 1 and 0.5, 1, 1.5, and 2×10^−2^ pA/µm^2^ for Figure 3 and 6.
3. **Currents evoked by a fixed action potential waveform at different temperatures.** To examine the effects of changes of action potential duration on the inward Na^+^ currents and the overlap of Na^+^ and K^+^ currents, we changed the cortical axon model such that the membrane potential was moved through the action potential trajectory obtained at either 18°C or 37°C, while the kinetics of the ion channels were varied between 6 and 37°C, according to the equations above. During these simulations, the time step was set to 10 µs, and the normal interaction of current entry and change in membrane potential was removed.
4. **Oxygen measurements and alteration in vitro.** To examine the influence of changes in oxygenation on the effects of changing temperature on neuronal firing rate, we first measured the oxygen content (along with neuronal responsiveness to intrasomatic injection of current pulses) within the cortical slice at temperatures varying from 30–42°C. Following this, we then independently manipulated oxygen content, while keeping temperature constant at 30°C and examined the effect of changes in oxygen concentration on neuronal responsiveness. The oxygen concentration was measured at 1 Hz in acute slices of mouse entorhinal cortex (12–18 days) using an OxyLite system (Oxford Optronix, Oxford, UK). The oxygen probe (Model BF/OT/E), which has a tip diameter of 350 µm, was placed 100 µm beneath the surface of the tissue in layer 2/3 of entorhinal cortex. Each temperature change was brought to steady state for at least two minutes before the response of the pyramidal neuron to intrasomatic injection of a depolarizing current pulse was measured. In additional experiments, we manipulated oxygen concentration while keeping temperature constant by bubbling the solution with varying proportions of O_2_/CO_2_ (95%/5%) and N_2_/CO_2_ (95%/5%) gas. Each oxygen level was maintained for at least two minutes before the firing rate was measured.

## Supporting Information

Figure S1 Relationship between excess Na^+^ entry ratio and temperature under control conditions (black), and when the Hodgkin-Huxley model of a cortical axon is run with an action potential wave form that is fixed to that occurring at 18°C (green) and 37°C (red). Note that in both experimental cases, using the fixed action potential waveform at colder temperatures results in a large increase in excess Na^+^ entry ratio, even though these spikes are shorter in duration than those that would have occurred at these colder temperatures. These results indicate that the decrease in Na^+^ entry ratio with temperature seen in control is not due to the shortening of the action potential duration.
(TIF)

Figure S2 A–C. Phase plots of membrane potential V vs. Na^+^ activation variable m (A), Na^+^ inactivation variable h (B) and K^+^ activation variable n (C) for temperature T = 18°C, and 36°C, respectively. Also plotted (black traces) are the steady state values of these variables (m_inf; h_inf; n_inf). Note that although increasing temperature does increase the resemblance of the m versus V phase plot to the steady state relation, the same is not as true for Na^+^ channel inactivation (h) or K^+^ channel activation (n). Here, the phase plots still exhibit substantial deviations from steady state values during action potential generation.
(TIF)

Figure S3 Increasing temperature increases firing rate during spontaneous Up states in entorhinal cortical slices. A. Example of an Up-state generated spontaneously in the medial entorhinal cortex at 23°C. B. Up states at 36°C. C. Up states at 42°C. Note the increase in Up rate, and increase in action potential discharge during each Up state. D–F. Action potential discharge rate (D), action potential duration (E), spike threshold (F) for action potentials occurring during Up states as a function of temperature. G. Resting membrane potential during the Down state at different temperatures. All data obtained from layer 2/3 pyramidal neurons (n = 6) in the mouse medial entorhinal cortex.
(TIF)

Figure S4 Effects of changing specific membrane resistance (decrease by 35%) and membrane potential (depolarization by 7 mV) on the relationship between temperature and firing rate in a HH model neuron (A) and the resulting effect on total Na^+^ charge entry in response to a DC pulse (B).
(TIF)

Figure S5 Increases in Na^+^ entry owing to changes in apparent input resistance and membrane time constant in a model neuron. A. Increasing specific membrane resistance from 20,000 to 60,000 only slightly decreases the excess Na^+^ entry ratio. B, C. Example action potentials with a specific membrane resistance of 18,000 and 66,000 Ohms. D, Relationship between membrane time constant and input resistance in the model. E. Excess Na^+^ entry ratio decreases only slightly by increasing membrane time constant. F. Firing rate of the neuron to a constant current pulse exhibits a small increase with a large increase input resistance.
(TIF)
