# Supplementary figures and images for: Warm Body Temperature Facilitates Energy Efficient Cortical Action Potentials
Source: PLoS Comput Biol. 2012 Apr 12;8(4):e1002456. doi: 10.1371/journal.pcbi.1002456 (PMC3325181; doi:10.1371/journal.pcbi.1002456)

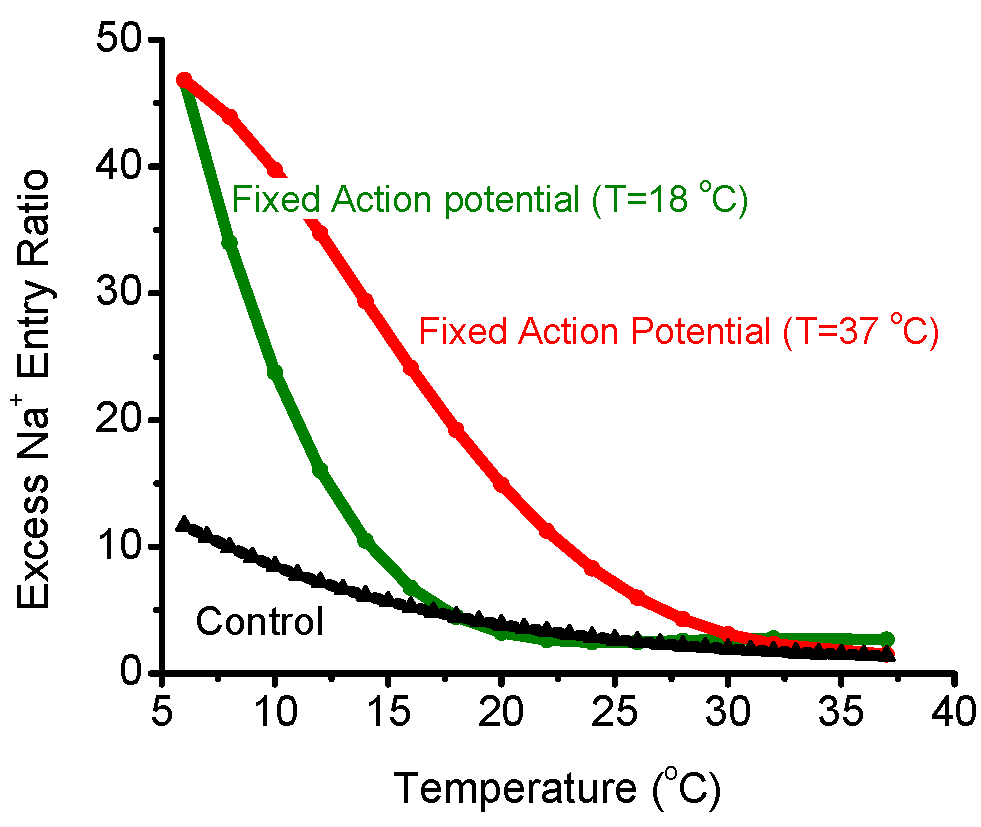

Supplement: Figure S1 — Relationship between excess Na+ entry ratio and temperature under control conditions (black), and when the Hodgkin-Huxley model of a cortical axon is run with an action potential wave form that is fixed to that occurring at 18°C (green) and 37°C (red). Note that in both experimental cases, using the fixed action potential waveform at colder temperatures results in a large increase in excess Na+ entry ratio, even though these spikes are shorter in duration than those that would have occurred at these colder temperatures. These results indicate that the decrease in Na+ entry ratio with temperature seen in control is not due to the shortening of the action potential duration. (TIF) [file pcbi.1002456.s001.tif]

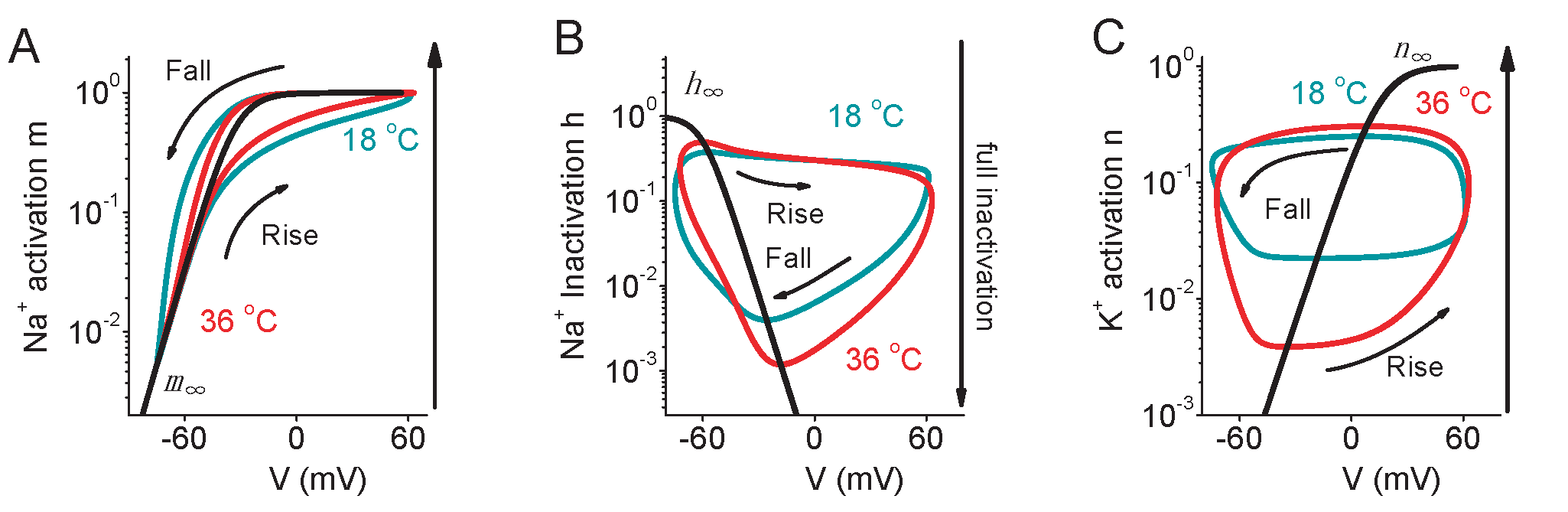

Supplement: Figure S2 — A–C. Phase plots of membrane potential V vs. Na+ activation variable m (A), Na+ inactivation variable h (B) and K+ activation variable n (C) for temperature T = 18°C, and 36°C, respectively. Also plotted (black traces) are the steady state values of these variables (m_inf; h_inf; n_inf). Note that although increasing temperature does increase the resemblance of the m versus V phase plot to the steady state relation, the same is not as true for Na+ channel inactivation (h) or K+ channel activation (n). Here, the phase plots still exhibit substantial deviations from steady state values during action potential generation. (TIF) [file pcbi.1002456.s002.tif]

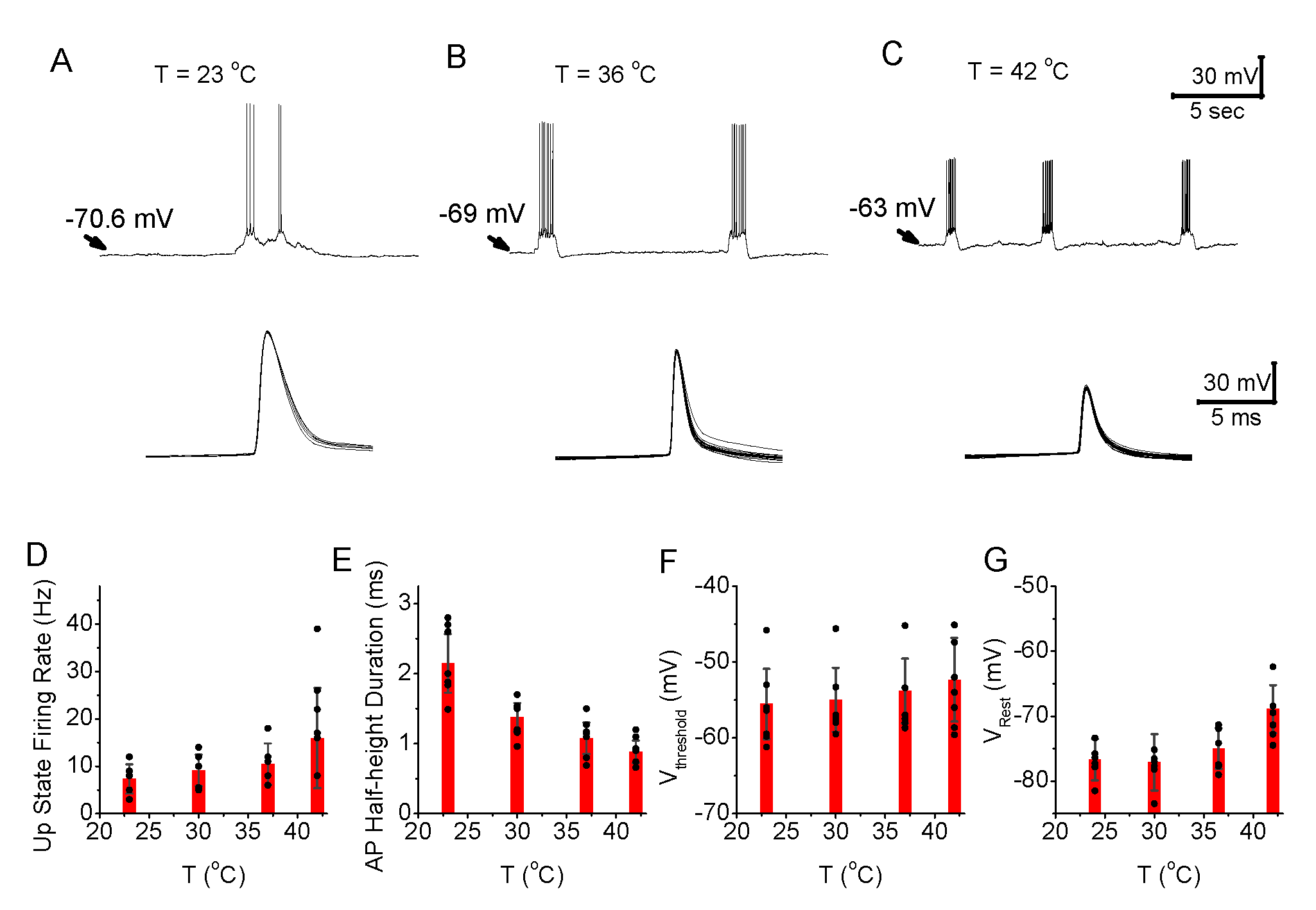

Supplement: Figure S3 — Increasing temperature increases firing rate during spontaneous Up states in entorhinal cortical slices. A. Example of an Up-state generated spontaneously in the medial entorhinal cortex at 23°C. B. Up states at 36°C. C. Up states at 42°C. Note the increase in Up rate, and increase in action potential discharge during each Up state. D–F. Action potential discharge rate (D), action potential duration (E), spike threshold (F) for action potentials occurring during Up states as a function of temperature. G. Resting membrane potential during the Down state at different temperatures. All data obtained from layer 2/3 pyramidal neurons (n = 6) in the mouse medial entorhinal cortex. (TIF) [file pcbi.1002456.s003.tif]

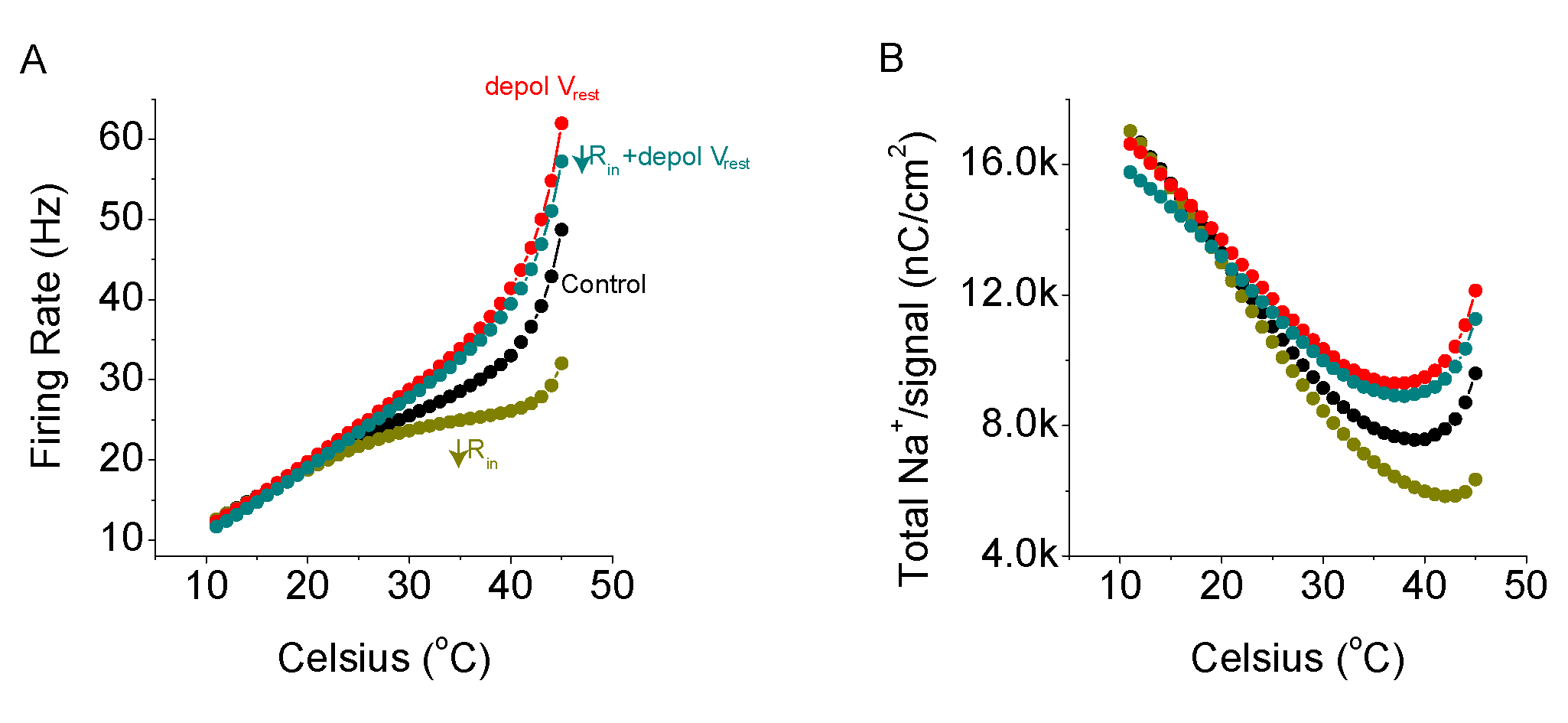

Supplement: Figure S4 — Effects of changing specific membrane resistance (decrease by 35%) and membrane potential (depolarization by 7 mV) on the relationship between temperature and firing rate in a HH model neuron (A) and the resulting effect on total Na+ charge entry in response to a DC pulse (B). (TIF) [file pcbi.1002456.s004.tif]

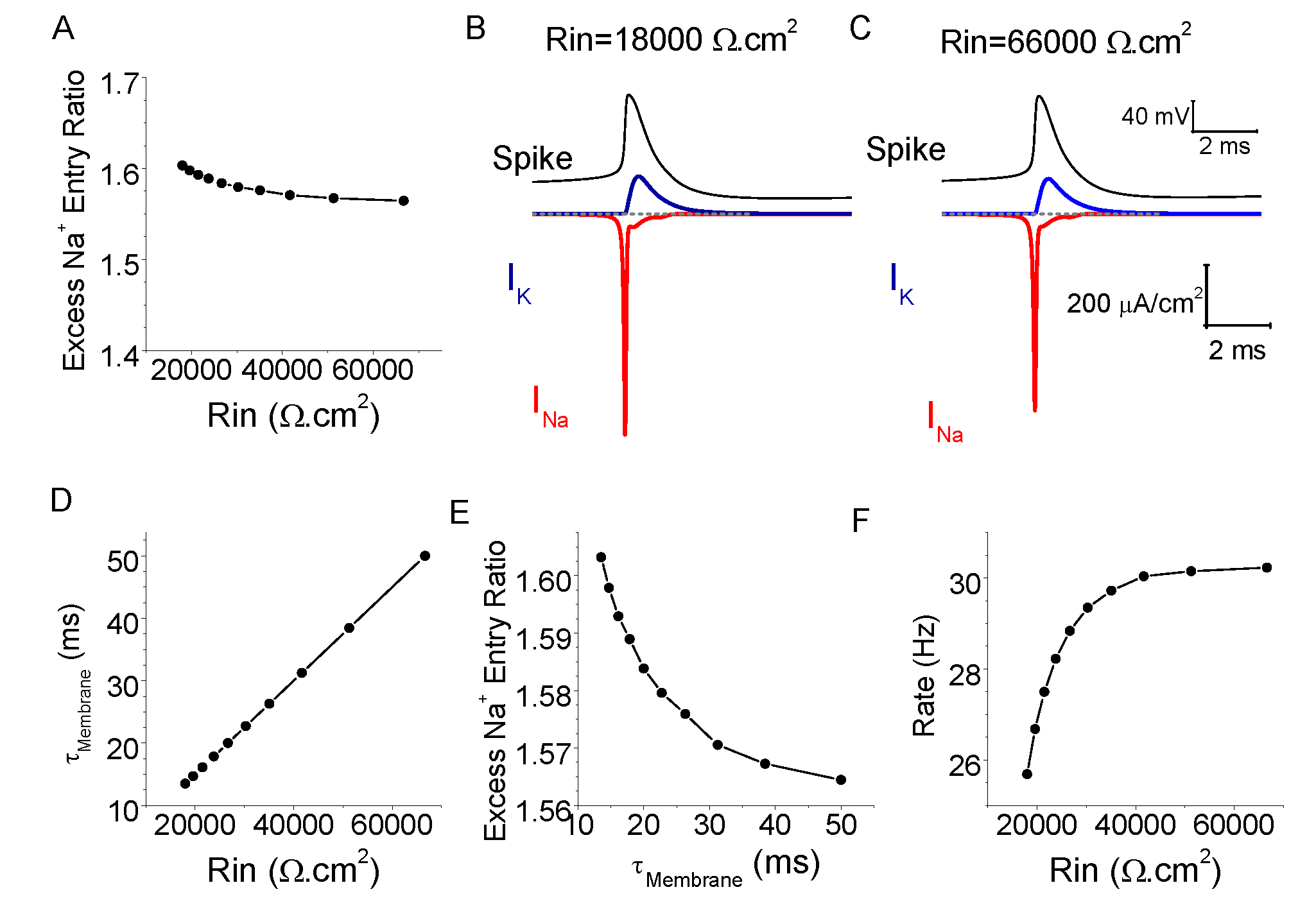

Supplement: Figure S5 — Increases in Na+ entry owing to changes in apparent input resistance and membrane time constant in a model neuron. A. Increasing specific membrane resistance from 20,000 to 60,000 only slightly decreases the excess Na+ entry ratio. B, C. Example action potentials with a specific membrane resistance of 18,000 and 66,000 Ohms. D, Relationship between membrane time constant and input resistance in the model. E. Excess Na+ entry ratio decreases only slightly by increasing membrane time constant. F. Firing rate of the neuron to a constant current pulse exhibits a small increase with a large increase input resistance. (TIF) [file pcbi.1002456.s005.tif]
